# Integrated analysis of single-cell RNA-seq and bulk RNA-seq unveils heterogeneity and establishes a novel signature for prognosis and tumor immune microenvironment in ovarian cancer

**DOI:** 10.1186/s13048-022-01074-1

**Published:** 2023-01-16

**Authors:** Zitao Wang, Jie Zhang, Fangfang Dai, Bingshu Li, Yanxiang Cheng

**Affiliations:** Department of Obstetrics and Gynecology, Renmin Hospital of Wuhan University, Wuhan, Hubei China

**Keywords:** Ovarian cancer, Single cell RNA-seq, Differentiation trajectory, Signature, Tumor immune microenvironment

## Abstract

Ovarian cancer is a highly heterogeneous gynecological malignancy that seriously affects the survival and prognosis of female patients. Single-cell sequencing and transcriptome analysis can effectively characterize tumor heterogeneity to better study the mechanism of occurrence and development. In this study, we identified differentially expressed genes with different differentiation outcomes of tumor cells by analyzing a single-cell dataset. Based on the differentially expressed genes, we explored the differences in function and tumor microenvironment among clusters via consensus clustering. Meanwhile, WGCNA was employed to obtain key genes related to ovarian cancer. On the basis of the TCGA and GEO datasets, we constructed a risk model consisting of 7 genes using the LASSO regression model, and successfully verified that the model was characterized as an independent prognostic factor, efficiently predicting the survival prognosis of patients. In addition, immune signature analysis showed that patients in the high-risk group exhibited lower anti-tumor immune cell infiltration and immunosuppressive status, and had poorer responsiveness to chemotherapeutic drugs and immunotherapy. In conclusion, our study provided a 7-gene prognostic model based on the heterogeneity of OC cells for ovarian cancer patients, which could effectively predict the prognosis of patients and identify the immune microenvironment status of patients.

**Supplementary Information:**

The online version contains supplementary material available at 10.1186/s13048-022-01074-1.

## Introduction

Ovarian cancer (OC) is the deadliest disease among gynecological malignancies. Due to the lack of representative symptoms, sensitive screening and diagnostic methods in the early stage, more than 70% of patients with OC are diagnosed at a late stage. Surgery, radiotherapy and chemotherapy are currently the main clinical treatments for OC. Although this combination therapy has improved patients’ outcomes, the 5-year survival rate of patients with late stage is still less than 30% owing to frequent recurrence and poor prognosis [1]. Besides, OC is a heterogeneous disease with major genetic events whose biological behavior is influenced by complex gene regulatory networks. There are several histological subtypes of aggressive OC, and each subtype is associated with distinct genetic and epidemiological risk factors, clinical features, and possible cellular origin. Therefore, traditional pathological classification and clinically relevant genomic stratification are no longer sufficient to explain the heterogeneity of OC. Instead, in-depth exploration at the molecular level is needed to make accurate diagnosis, treatment, and prognosis. Hence, analysis of key genes based on cellular heterogeneity can provide potential immunotherapeutic targets and meaningful risk prediction for OC.

Single-cell RNA sequencing (scRNA-seq), which uses optimized next-generation sequencing technologies to define the global gene expression profile of a single cell, helps dissect previously hidden heterogeneity in cell populations, providing an efficient way to explore tumor heterogeneity and evolutionary mechanisms, and a better understanding of the underlying mechanisms of the disease and individualized treatment and an opportunity to comprehensively characterize genetic complexity at the cellular level, including differences in single nucleotide polymorphisms, copy number variation, gene expression levels, genomic structural variation, gene fusions, alternative splicing, and DNA methylation, further contributing to our understanding of cellular heterogeneity [2–4]. Recently, scRNA-seq of 13,571 cells from two OC patients reported the identification of cell populations and key pathways and genes that played important roles in the development of ovarian cancer cells [5]. Another study describing single-cell RNA profiles of six tumors also revealed extensive heterogeneity in OC [6]. Characterization of heterogeneous signatures within OC could surely lead to a better understanding of the mechanisms of ovarian carcinogenesis, metastasis and drug resistance.

Therefore, in this study, we performed a detailed biological analysis based on single-cell RNA-seq data from the GEO database and expression profiling data from bulk RNA-seq in The Cancer Genome Atlas (TCGA) and Gene Expression Omnibus (GEO) to identify differences between different cell clusters in OC. Based on the identified heterogeneity of OC cells, a prognostic risk model consisting of seven characteristic genes was constructed and validated, providing new biomarkers and potential therapeutic targets for the identification of heterogeneity and future targeted therapy.

## Materials and methods

### Data acquisition and processing

The scRNA-seq data in GSE118828 and bulk-seq data in GSE138876 and the corresponding clinical information were obtained from the GEO database, and the TCGA-OV RNA-seq data and the corresponding clinical data were obtained from TCGA, in which the TCGA dataset was used as the training set, and the GSE138876 was used as external validation dataset. The SVA package was used to normalize TCGA and GEO data to remove batch effects.

### Acquisition and processing of scRNA-seq and bulk RNA-seq

scRNA-seq data for 17 ovarian cancer samples and 1 normal tissue sample were obtained from the GSE118828 dataset in the GEO (https://www.ncbi.nlm.nih.gov/geo/) database. The scRNA-seq data were first processed by the Seurat package, the percentage of mitochondrial genes was calculated by the PercentageFeatureSet function, and the relationship between sequencing depth and mitochondrial gene sequence and/or total intracellular sequence was elucidated by correlation analysis. Cells with gene count < 100, sequence count < 50, and mitochondrial gene content > 5% were excluded. After data filtering, the scRNA-seq data were normalized by the LogNormalize method. t-SNE principal component analysis (PCA) was used to perform unsupervised clustering and unbiased visualization of cell populations on 2D maps. Subsequently, marker genes for each cluster were identified using the “FindAllMarkers” function. The SingleR package was used for marker-based cell type annotation. Finally, the “Monocle” package was utilized to perform pseudo-time and trajectory analysis of cells, and the differentially expressed genes between different trajectories were screened with the criteria of FC = 1, adj *P* value = 0.05, in which “fdr” functioned as *P* value adjustment method. The “clusterProfiler”, “org.Hs.eg.db”, “enrichplot” and “ggplot2” packages were used for Gene Ontology (GO) annotation and Kyoto Encyclopedia of Genes and Genomes (KEGG) enrichment analysis.

### Consensus clustering based on differentially expressed genes (DEGs)

The “ConsensusClusterPlus” package provided quantitative and visual evidence of stability for measuring the number of unsupervised subtypes for consensus clustering of OCs. The optimal number of subtypes was determined using the K-means algorithm and cumulative distribution function curves, with 50 iterations for stable subtypes, in which max K = 9. Kaplan-Meier analysis was completed to assess survival outcomes and the proportion of clinicopathological features in each molecular subtype was displayed using the “ggplot2” package.

### Tumor immune microenvironment

To investigate the immune microenvironment composition of each patient, the “ESTIMATE” package calculated the immune, stromal score and tumor purity for each sample. The content of 22 immune cells in each sample was identified by CIBERSORT software, and the relative immune cell infiltration levels of individual samples were quantified by single-sample gene set enrichment analysis (ssGSEA) in the R package gsva. Meanwhile, the immune cell infiltration content of different subtypes was compared through the “limma” package, showing a significant difference in immune cell histograms.

### WGCNA

Based on the DEGs identified in single-cell sequencing, the R package “WGCNA” was used to construct a co-expression network of DEGs. In addition, the Pearson correlation coefficient of the genes was calculated to obtain a similarity matrix. In order to make the network conform to the scale-free network distribution, appropriate weight values and soft threshold were chosen and calculated to measure the connectivity between genes and the adjacency matrix was converted to a topological overlap matrix. Besides, the matrix was hierarchically clustered using the WGCNA package. In terms of the generated clustering tree, the Dynamic Tree Cut method was used to cut the gene clustering tree. Genes with similar expression patterns were assigned to a branch, each branch representing a co-expression module. Correlations between genes in each module and clinical features were determined by gene significance and module membership. The identified genes were differentially analyzed using the limma package with the criteria of FC = 1, adj *P* = 0.05, and the “ggplot2” and “pheatmap” packages were used to visualize the DEGs.

### Construction and validation of the risk model

To evaluate the prognostic value of the key genes, we first performed univariate COX analysis on the identified DEGs to explore their correlation with survival status, and identified 11 prognostic-related genes for further analysis. LASSO Cox regression model (R package “glmnet”) was then used to narrow down the candidate genes and develop a prognostic model. Finally, 7 genes and their coefficients were retained, and the penalty parameter (λ) was determined by the minimum criterion. The risk score was calculated after normalizing the TCGA expression dataset (applying the “scale” function in R), and the risk score formula was as follows: risk score = ∑7iXi × Yi (X: coefficient, Y: gene expression grade). OC patients in TCGA were divided into low-risk and high-risk subgroups according to the median risk score, and the OS time between the two subgroups was compared by Kaplan-Meier analysis. PCA and tSNE based on the 7-gene signature was performed by the “prcomp” function in the “stats” R package. The “survival”, “survminer” and “timeROC” R packages were employed to perform 3-year ROC curve analysis. For the validation study, the OC cohort from the GEO database (GSE138876) was performed.

### Independent prognostic analysis

To investigate the relationship between risk score and clinical characteristics, risk score was integrated with clinical information and analyzed via univariate and multivariate COX regression models.

### Construction of the nomogram

Integrating risk score and clinical parameters together, the rms package was used to construct a nomogram to predict the survival prognosis of patients at 1, 3 and 5 years, and calibration curves were generated to verify the performance of the nomogram.

### Chemotherapeutic drug sensitivity and immunotherapy responsiveness

To predict chemosensitivity between high and low risk groups, we employed the pRRophetic R package to infer common chemotherapeutic maximal inhibitory concentration (IC50) values by building a ridge regression model with 10-fold cross-validation. Using immune checkpoint molecules to assess patients’ potential response to immunotherapy. At the same time, TIDE was a computational method that simulated two major mechanisms of tumor immune escape T-cell dysfunction and T-cell rejection, which could be employed to predict the prognosis of the patients to immune checkpoint inhibitor response. Eventually, TIDE scores were calculated and compared between high and low risk groups to assess patient responsiveness to immunotherapy.

### Mutation analysis

Mutation data for OC patients were also obtained from the TCGA data portal (https://portal.gdc.cancer.gov/). The data were further analyzed using the “maftools” package. We calculated the tumor mutational burden (TMB) score for each patient as follows: (total mutations ÷ total bases covered) × 10^6. Meanwhile, gene mutation profiles of model genes were searched on the cBioportal website (www.cBioportal.org/).

### qRT-PCR validation of the expression of the model genes

Three pairs of matched OC and para-carcinoma tissue were obtained from the Gynecology and Obstetrics in Renmin Hospital of Wuhan University. All the patients provided informed consent and were approved by the Ethics Committee of Renmin Hospital of Wuhan University. Total RNA from OC and para-carcinoma tissue samples were extracted and quantitated by qRT-PCR, where GAPDH was used as an internal control.

## Results

### Quality control and normalization of scRNA-seq data

**Fig. 1:**
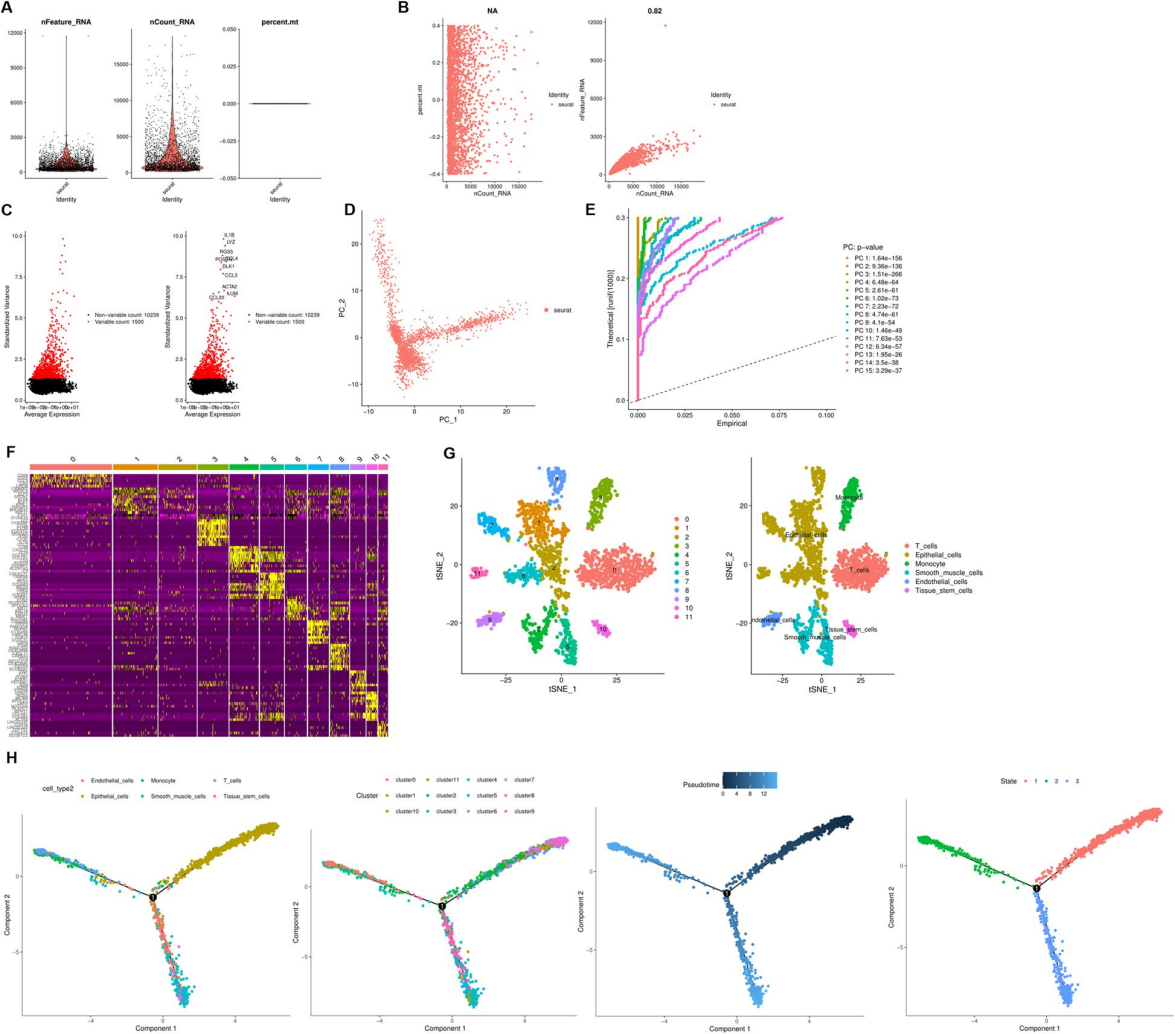
Analysis of single-cell RNA sequencing. **A** Post quality control filtering of each sequenced cell, which was plotted in violin plots to display their number of RNA features (nFeature_RNA) and absolute UMI counts (nCount_RNA). **B** Correlation analysis between sequencing depth and mitochondrial gene sequences and total intracellular sequences. **C** 10239 non-variable genes and 1500 variable genes were analyzed. **D** PCA based on scRNA-seq data. **E** 15 PCs were identified as the criteria of *P* < 0.5. **F** Heatmap illustrated the expression patterns of the top ten markers in individual cells of each cluster by Seurat analysis. **G** Cells were clustered into 12 types via tSNE analysis. **H** Pseudotime and trajectory analysis

In this study, 16 tumor samples and 1 normal tissue sample were obtained from GSE118828 (An ineligible tumor sample was excluded). A total of 16,383 transcripts in 355 single cells were preprocessed for scRNA sequencing analysis. After filtering for the total number of genes expressed in individual cells and the percentage of mitochondrial reads, no correlation between sequencing depth and mitochondrial gene sequence was detected (Fig. 1A). Sequencing depth was significantly positively correlated with total intracellular sequences (R = 0.82, Fig. 1B). A total of 11,739 genes were analyzed, of which 10,239 had low cell-to-cell variation and 1500 had high variation (Fig. 1C).

### Single-cell RNA-seq analysis, clustering, cell trajectory analysis

PCA was used for preliminary dimensionality reduction of scRNA-seq data. Unbiased PCA was performed on 355 cells using highly variable genes to examine global transcriptome patterns in scRNA-seq data. The results showed that there was no obvious separation between cells (Fig. 1D), then the top 15 principal components with significant differences were selected for further analysis (Fig. 1E). According to tSNE algorithm, 355 cells were aggregated into 12 clusters, and a total of 1738 marker genes were identified by differential analysis. The top 10% marker genes in each cluster are shown in the heatmap (Fig. 1F). Twelve clusters were annotated according to marker genes: cluster 0 was closely related to T cells, 1 2 6 7 8 11 was closely related to epithelial cells, cluster 3 was closely related to monocytes, cluster 9 was closely related to endothelial cells, and clusters 4 5 were closely related to Smooth muscle cells, cluster 10 are closely related to tissue stem cells (Fig. 1G). To determine the relationship between these cell clusters and states, differentiation trajectories and pseudo-temporal analyses were studied using the Monocle2 R package based on the identified marker genes from each cluster. According to the pseudo-time order, the cells seem to start with cluster 2 and 9, transition to cluster 3, 6 and 7, and finally to cluster 0, 4 and 8 (Fig. 1H). Not only that, we could find that cluster 2, 6, 9 and 11 were distributed in state 1, cluster 0 and 4 were distributed in state 2, while cluster 3, 5 and 10 were distributed in state 3, indicating different differentiation progression outcomes (Fig. 1H). Except for above analysis results, we also integrated a GEO cohort (GSE154600) for validation, which indicated that there were different cell clusters in all cells, such as T cells, Fibroblasts, Tissue stem cells, etc. (Fig. S1).

### Functional enrichment analysis

In order to understand the differences in different differentiation outcomes of cells, we used Limma to perform differential analysis to identify DEGs (logFC = 1, adj *P* value = 0.05). There were 371 DEGs in state 2, 491 DEGs in state 2 and 332 DEGs in state 3. To further identify the enrichment functions of DEGs, we performed GO and KEGG enrichment analyses. GO analysis showed that state 1 and state 2 were enriched in negative regulation of cell adhesion, cell adhesion mediator activity, regulation of cell-cell adhesion; State 3 was enriched in extracellular matrix organization, collagen-containing extracellular matrix, and extracellular matrix structural constituent (Fig. S2A). KEGG enrichment analysis found that state1, 2 and 3 were enriched in PI3K-Akt signaling pathway, ECM-receptor interaction, and Focal adhesion (Fig. S2B).

### Consensus cluster analysis

**Fig. 2:**
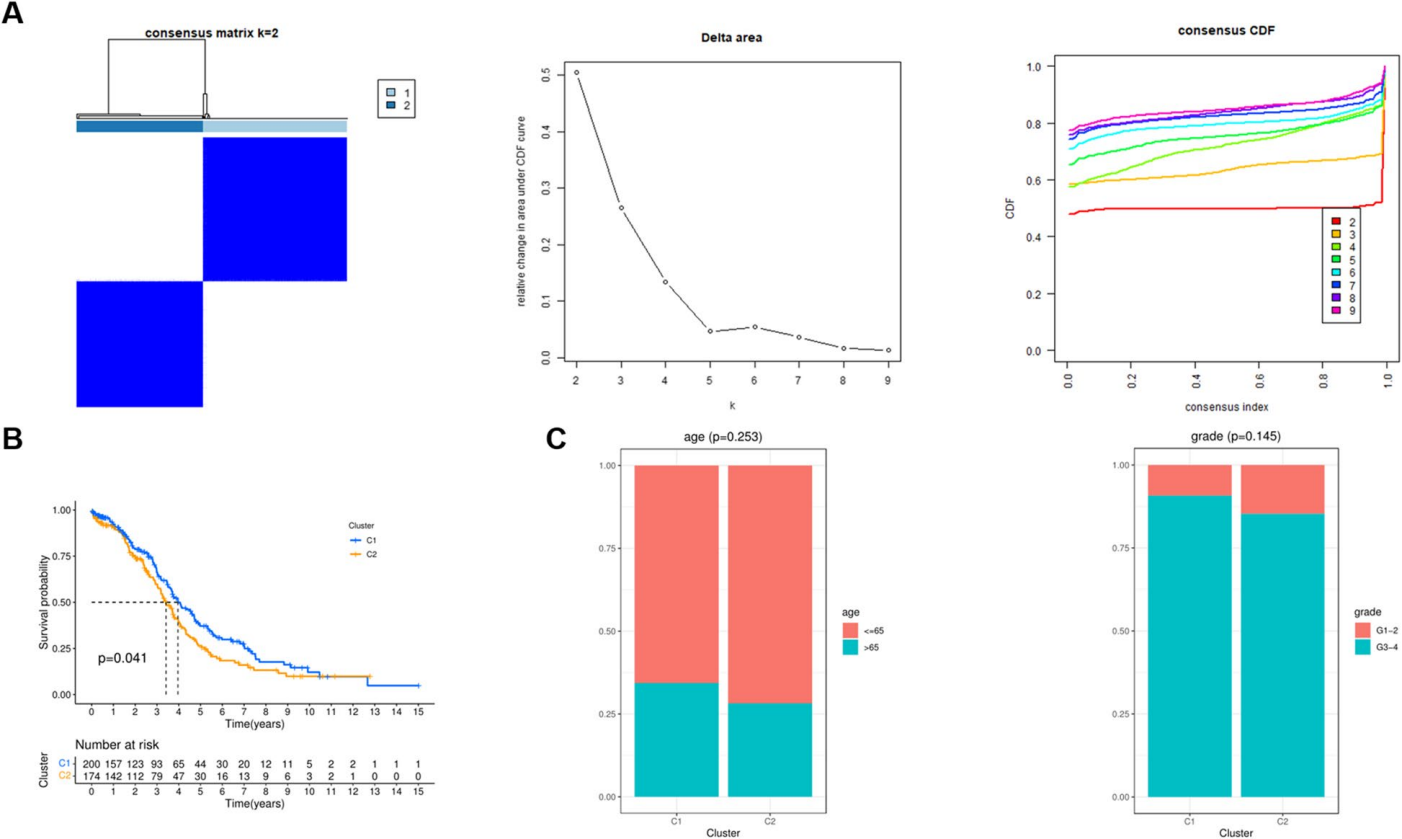
Consensus cluster analysis based on the hub genes. **A** Two clusters were identified at a clustering threshold of K = 9. **B** Kaplan-Meier analysis between the two clusters. **C** Correlation analysis between clusters and clinicopathologic features

To better investigate the mechanism of occurrence and development of OC, consensus clustering analysis was performed to classify all patients in TCGA into clusters. Based on gene expression profiles and survival time, we found that when the clustering was 2, patients could be well classified (Fig. 2A). Further Kaplan-Meier (KM) analysis found that C1 patients had better survival prognosis than C2 patients (Fig. 2B). Then we combined the clinical characteristics of the patients, such as age, grade and grouping, and correlation analysis found that there was no significant difference in age distribution and pathological grade between the C1 and C2 groups (Fig. 2C).

### The immune microenvironment among different clusters

**Fig. 3:**
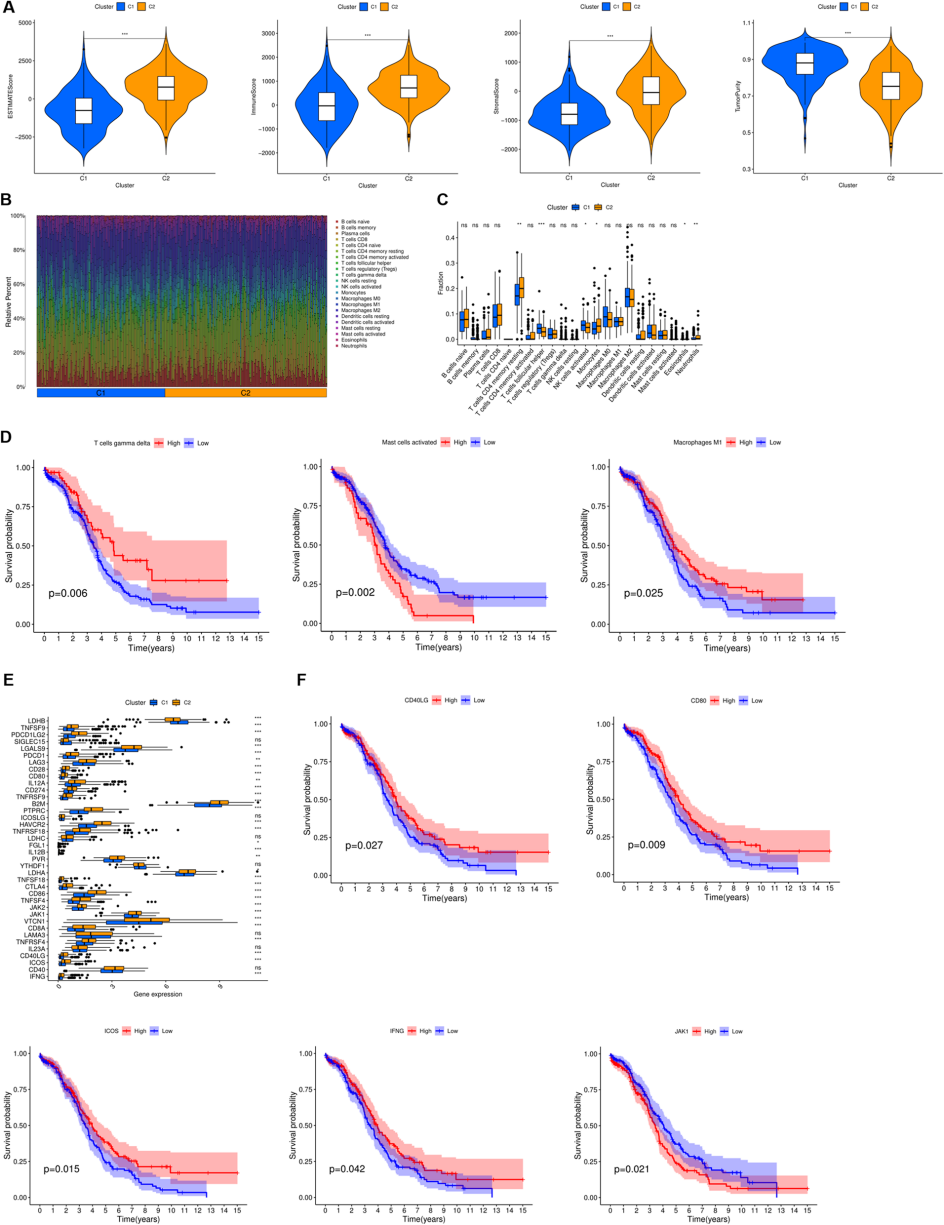
The immune microenvironment among different clusters. **A** Tumor microenvironment scores between clusters. **B** Heatmap illustrated the contents of 22 immune cells in each sample. **C** Comparison of 22 immune cells between clusters. **D** Kaplan-Meier analysis of immune cells. **E** Comparison of immune checkpoint molecules between clusters. **F** Kaplan-Meier analysis of immune checkpoint molecules

CIBERSORT was utilized to calculate tumor microenvironment immune score of each patient and we therefore calculated and compared immune scores in different clusters of patients. The violin plot clearly showed that the Estimate score, Immune score, and Stromal score in the C1 group were significantly lower than those in the C2 group, while the Tumor purity was significantly higher than that in the C2 cluster (Fig. 3A). The content of 22 immune cells in each sample was calculated according to the CIBERSORT algorithm and displayed visually with different colors representing different cell types (Fig. 3B). Difference analysis showed that T cells CD4 memory resting, Monocytes, Eosinophil and Neutrophils were significantly higher in C2 cluster than C1 cluster, which were closely associated with poor prognosis, while T cells follicular helper, NK cells activated were significantly increased in C1 cluster (Fig. 3C). We then investigated immune cells closely related to survival prognosis. The results showed that the prognosis of patients with high expression of T cell gamma delta (*P* = 0.006) and M1 macrophages (*P* = 0.002) was significantly better than that of patients with low expression, while the prognosis of patients with low expression of Mast cells activated was better than that of patients with high expression (*P* = 0.025). (Fig. 3D). Immune checkpoint molecules referred to key target molecules that inhibited the function of immune cells. Inhibiting these targets molecules could activate immune function, that was, immune checkpoint inhibitors. To assess the responsiveness of the clusters to immune checkpoint therapy, we calculated and compared the expression of immune checkpoint molecules between the two groups, and the results showed that the expression of *LDHB*, *TNFSF9*, *PDCD1LG2*, *LGALS9*, *PDCD1*, *LAG3* was significantly decreased in the C1 cluster, *CD28*, *CD80*, *IL12A*, *CD274*, *TNFRSF9*, *B2M*, *PTPRC*, *ICOSLG*, *HAVCR2*, *TNFRSF18*, *FGL1*, *IL12B*, *PVR*, *LDHA*, *TNFSF18*, *CTLA4*, *CD86*, *TN*FSF4, *JAK2*, *JAK1*, *VTCN1*, *CD8A*, *TNFRSF4*, *CD40LG*, *ICOS*, *IFNG* expression was significantly increased in the C2 cluster (Fig. 3E). More importantly, survival analysis found that *CD40LG*, *CD80*, *ICOS*, *IFNG* predicted better prognosis, while *JAK1* predicted poor prognosis (Fig. 3F).

### Establishment, evaluation and verification of predictive risk model

**Fig. 4:**
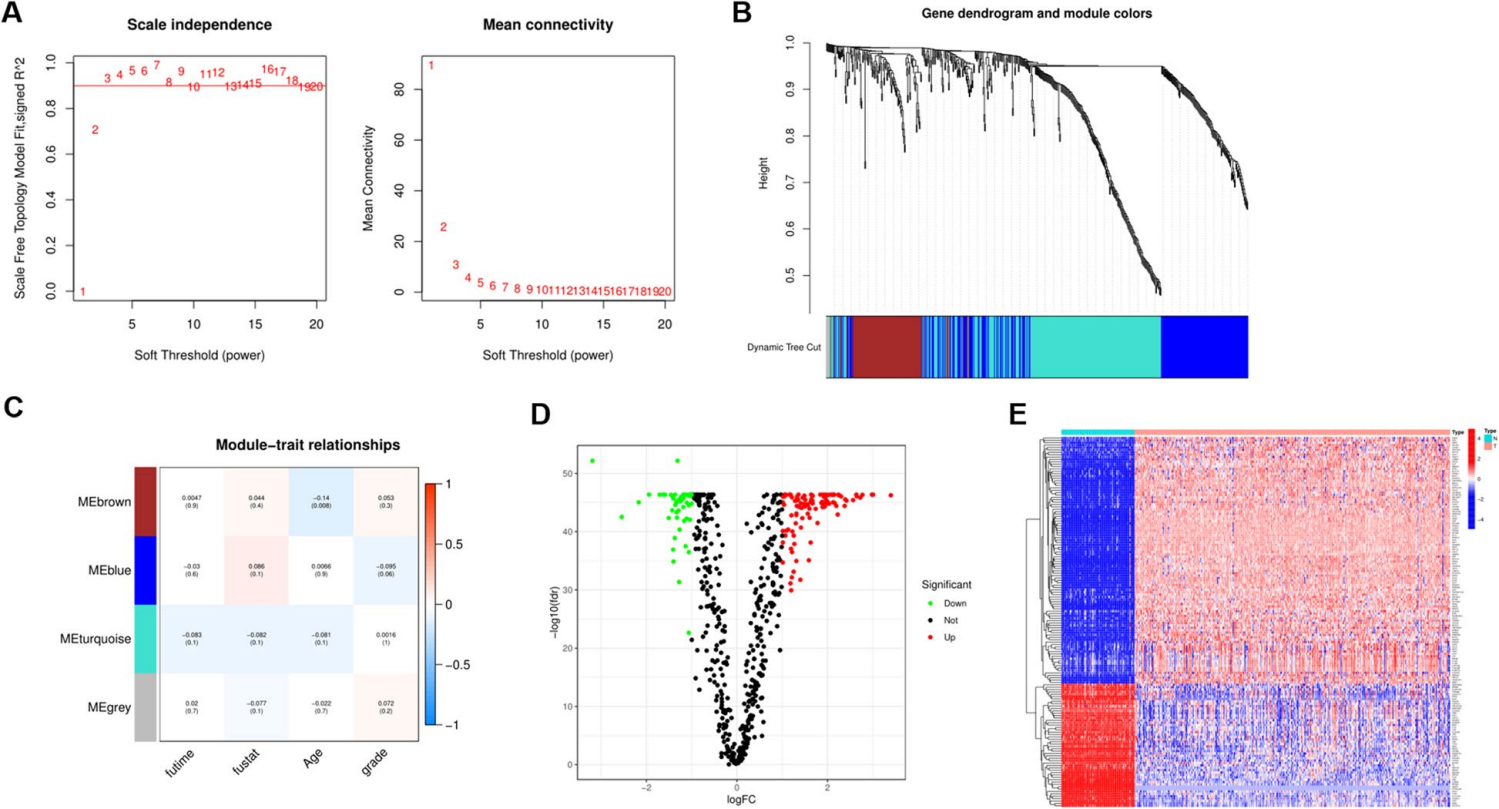
Identified hub genes for weighted correlation network analysis. **A-B** 4 modules were identified based on WGCNA analysis. **C** Correlation analysis between modules and clinicopathological parameters. **D** Differential expression analysis. **E** Heatmap illustrated the expression profiles of DEGs

**Fig. 5:**
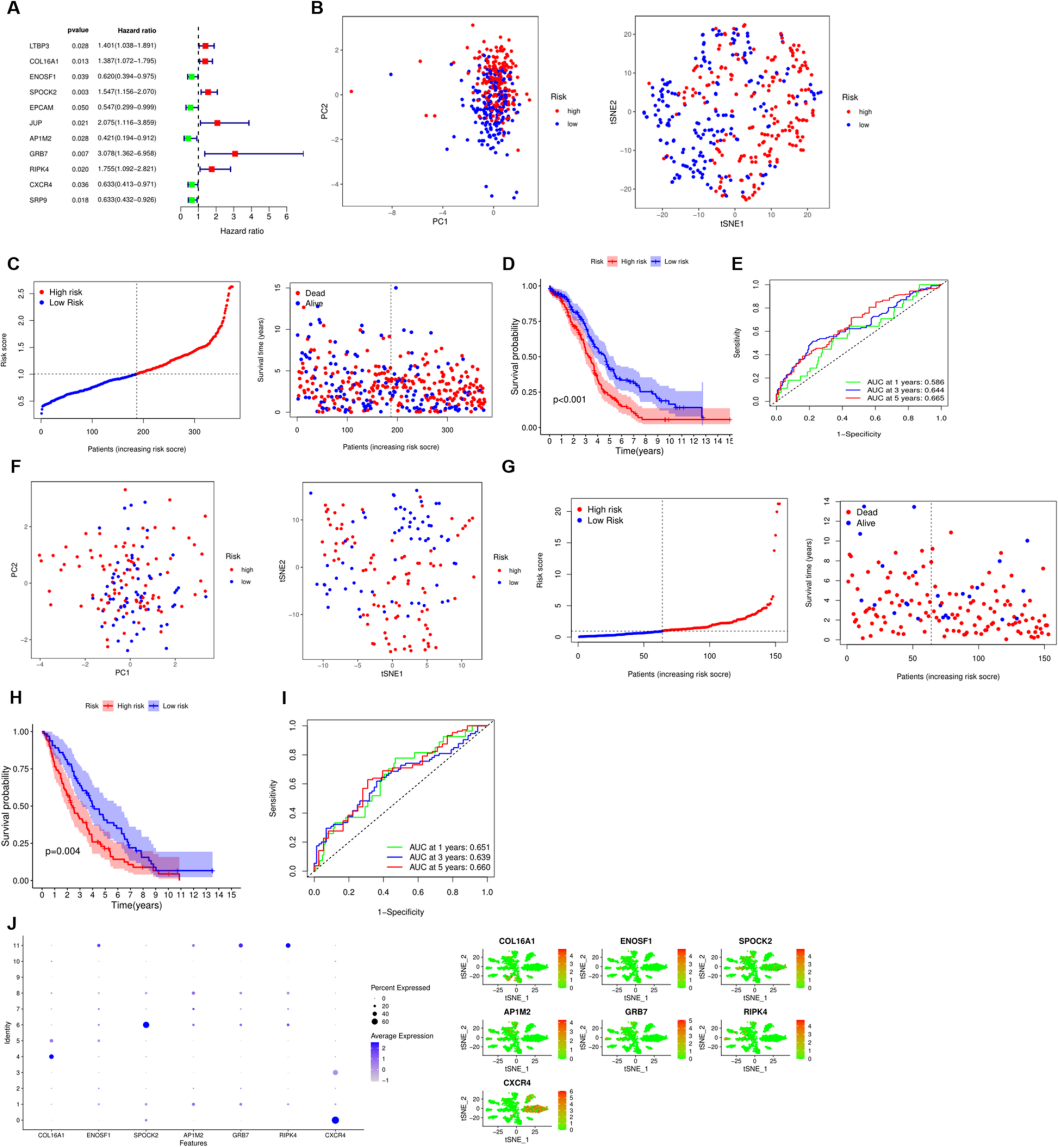
Construction and validation of the risk model. **A** Univariate analysis of prognosis-related DEGs. **B** PCA and tSNE analyses of patients in the training set. **C** Distribution of the risk score and survival status of patients in the training set. **D** Kaplan-Meier analysis between the low-risk group and the high-risk group in the training set. **E** ROC curves for predicting 1-year, 3-year and 5-year OS in the training set. **F** PCA and tSNE analyses of patients in the testing set. **G** Distribution of the risk score and survival status of patients in the testing set. **H** Kaplan-Meier analysis between the low-risk group and the high-risk group in the testing set. **I** ROC curves for predicting 1-year, 3-year and 5-year OS in the testing set. **J** Expression levels of 7 DEGs in 12 clusters

To construct a prognostic model for predicting the occurrence and development of OC, we newly incorporated the GEO dataset and performed WGCNA analysis based on the 719 DEGs by integrating the TCGA and GEO expression profiles (Fig. 4A, B). Clustering identified 4 modules such as blue, brown, cyan and grey modules (Fig. 4C). Then, differential expression analysis was performed according to the identified characteristic genes, and a total of 156 DEG were identified (FDR = 0.05 FC = 2), and the results were presented in the form of heatmap and volcano map (Fig. 4D, E). Subsequently, univariate COX analysis was used to identify key genes associated with prognosis, and the forest plot showed a total of 11 genes associated with prognosis, among which *LTBP3*, *COL16A1*, *SPOCK2*, *JUP*, *GRB7*, *RIPK4* were risk genes, *ENOSF1*, *EPCAM*, *AP1M2*, *CXCR4*, *SRP9* were protective genes (Fig. 5A). Multivariate COX analysis was employed to build the risk model based on prognostic genes. The risk model consisted of 7 core genes, and the formula was as follows: Risk score = (0.21319 * expression of *COL16A1*) + (− 0.42364 * expression of *ENOSF1*) + (0.30670 * expression of *SPOCK2*) + (− 1.24260 * expression of *AP1M2*) + (0.00013 * expression of *GRB7*) + (0.41622 * expression of *RIPK4*) + (− 0.56201 * expression of *CXCR4*). According to the risk score of the prognostic model, patients in TCGA and GEO were divided into two groups of high and low risk. PCA and tSNE analysis showed that the patients could be well differentiated (Fig. 5B). As the risk score gradually increased, the survival time of patients became shorter and the number of deaths increased (Fig. 5C). Survival analysis showed that the survival prognosis of patients in the low-risk group was significantly better than that of the high-risk patients (Fig. 5D). In addition, in the TCGA cohort, the areas under the receiver operating characteristic (ROC) curve for predicting 1-, 3-, and 5-year OS were 0.586, 0.644, and 0.665, respectively (Fig. 5E). Patients in the GEO dataset were classified into high- and low-risk groups based on the median risk score, and PCA and tSNE analyses confirmed the validity of their classification (Fig. 5F). The distribution of survival status also showed that an increase in the risk score was accompanied by a worsening of the patient’s survival status (Fig. 5G). Survival analysis further confirmed the better survival prognosis of patients in the low-risk group (Fig. 5H). The 1-year, 3-year, and 5-year values of AUC were 0.651, 0.639, and 0.660, respectively, verifying the high predictive ability of the risk model (Fig. 5I). In addition, the expression levels of 7 core genes in the 12 clusters are shown in Fig. 5J. *COL16A1* was increased in group 4; *ENOSF1*, *GRB7*, *RIPK4* were increased in cluster 11, *SPOCK2* was increased in cluster 6, *AP1M2* was increased in cluster 8, and *CXCR4* was significantly increased in cluster 0 (Fig. 5J). Subsequently, in order to better verify the predictive ability of the risk model, we included three data sets, including GSE23554, GSE26712 and GSE51088. The results clearly found that in the datasets, the survival prognosis of low-risk patients was significantly better than that of high-risk patients, and the AUC curves also clearly proved that the model had better prediction performance (Figs. S3, 4 and 5).

### Construction of the nomogram

We then integrated patient risk scores with clinical characteristics, and univariate and multivariate COX analyses in the TCGA dataset indicated that risk scores were independent risk factors in OC (Fig. S6A, B). Finally, we constructed a nomogram to predict the survival prognosis of patients, and the calibration curves illustrated the better performance of the nomogram (Fig. S6C).

### Comparison between the risk model and other established models

In order to evaluate the predictive ability of our model, we compared the C-index value of the model with other established models. We found that the C-index value of our model was 0.6, while the C-index of other models were 0.571, 0.591, and 0.56 respectively (Fig. S7A). At the same time, we compared the prediction performance of the nomogram. The DCA and AUC results also showed that the nomogram also had certain advantages compared with other clinical features (Fig. S7B, C).

### Independent prognostic analysis

To investigate the relationship between risk scores and clinical characteristics, we integrated patients’ clinical information with risk scores and presented them in the form of a heatmap (Fig. S8A). The correlation analysis was presented as a histogram (Fig. S8B). Finally, we performed a survival analysis, and the results showed that low-risk patients in < 65-year-old group, > 65-year-old group and the Stage 3-4 group were significantly better than the high-risk group, while the survival prognosis of the Stage1-2 group was not significantly different (Fig. S8C).

### Immune infiltration landscape in high and low risk groups

**Fig. 6:**
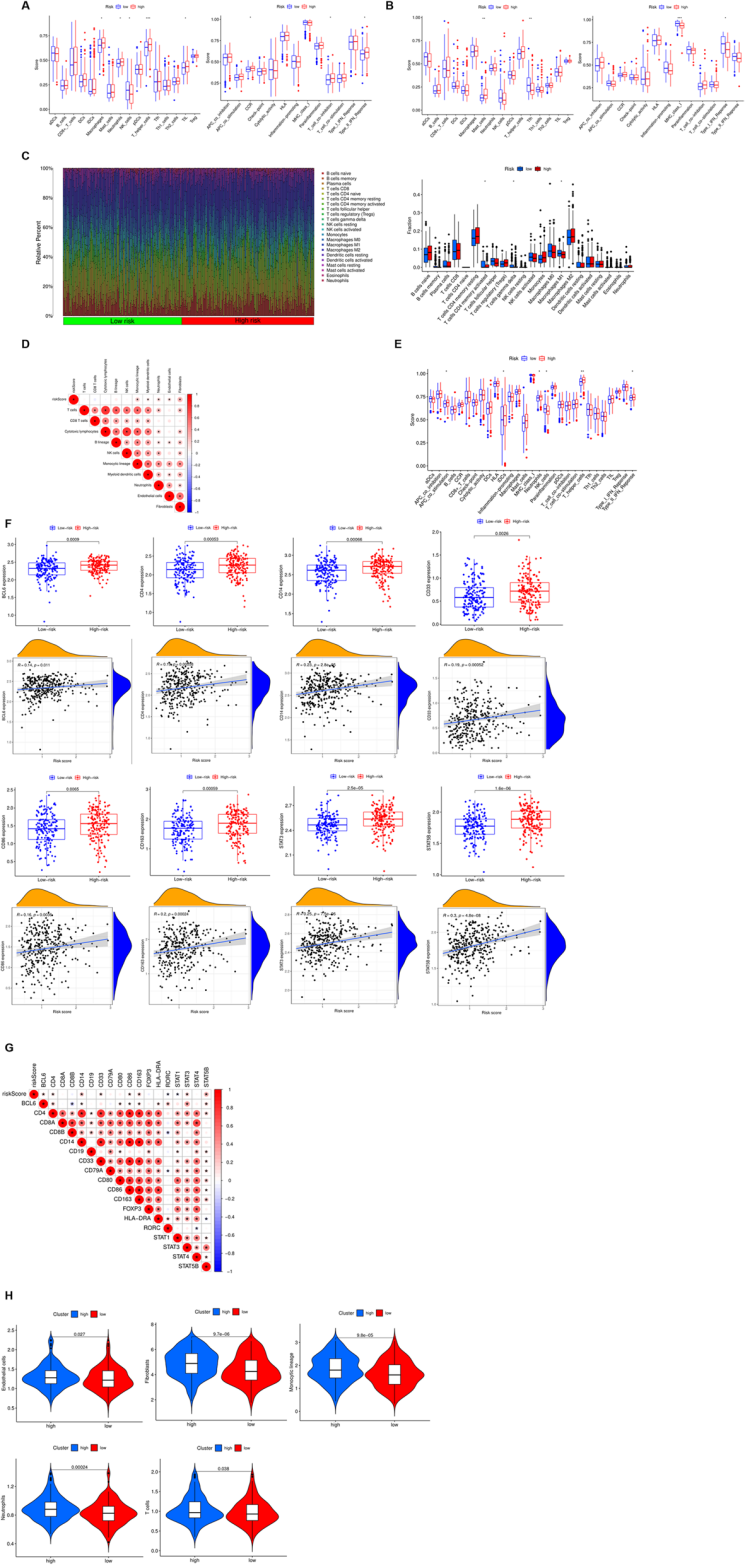
Immune infiltration landscape in high and low risk groups. **A B** Comparison of the immune cells and functions between the two groups in TCGA and GEO datasets. **C** Heatmap and infiltration of the immune cells between the two groups. **D** Correlation analysis between the risk score and immune cells. **E** Comparison of the Immune function between the two groups. **F** Comparison of the expression of the markers of immune cells between the two groups. **G** Correlation analysis between the risk score and immune cells markers. **H** Comparison of the immune cells and functions between the two groups in TCGA and GEO datasets via MCP-counter

ssGSEA was used to calculate the level of immune infiltration in each patient. By calculating and comparing the levels of immune cells in patients with high and low risk groups in the TCGA and GEO datasets, we found that the levels of Macrophages, Neutrophils, T helper cells, and TIL cells in the high- risk group were significantly higher than those in the low- risk group, while NK cells were significantly lower than those in the low risk group. The CCR, T cell co-inhibition, Type II IFN Response in the high-risk group of immune function were also significantly higher than those in the low-risk group (Fig. 6A). In the GEO dataset, Mast cells were significantly increased in the high-risk group, while Tfh, MHC class I, Type I IFN Response were significantly decreased (Fig. 6B). Not only that, we also used ESTIMATE to evaluate the relationship between risk models and immune cells. CIBERSORT results showed that T cells CD4 memory activated, T cells gamma delta, and Macrophages M1 expression were significantly increased in the low-risk group (Fig. 6C). Survival analysis showed that high M1 macrophages, Plasma cells, activated CD4^+^ T cells, Folicular helper T cells, gamma delta T cells had a significantly better prognosis than those with low expression, while M2 macrophages, activated Mast cells, Monocytes, Neutrophils, resting CD4^+^ T cells were opposite (Fig. S9). Correlation analysis also revealed a close relationship between various immune cells and risk scores (Fig. 6D). Immune function enrichment showed that APC co stimulation, iDCs, Neutrophils, T helper cell, Type II IFN Response were significantly enriched in the high-risk group, while NK cells were enriched in the low-risk group (Fig. 6E). Survival analysis also showed patients with high expression of aDCs, APC co inhibition prognosis had significantly better prognosis than those with low expression, and the prognosis of low expression of Type II IFN Response, iDCs, CCR, etc. was significantly better than that of high expression (Fig. S10). Not only that, we also identified the expression of specific markers in immune cells. The study found that the expression of *BCL6*, *CD4*, *CD14*, *CD33*, *CD86*, *CD163*, *STAT3*, *STAT5B* was significantly increased in the high-risk group, which was significantly positively correlated with the risk score, (Fig. 6F), which was further confirmed by Pearson correlation analysis (Fig. 6G). Eventually, the MCP-counter results demonstrated that patients with low risk had higher infiltration of endothelial cells, fibroblasts, monocytes, neutrophils and T cells (Fig. 6H).

### Risk score and immunotherapy and chemotherapy responsiveness

**Fig. 7:**
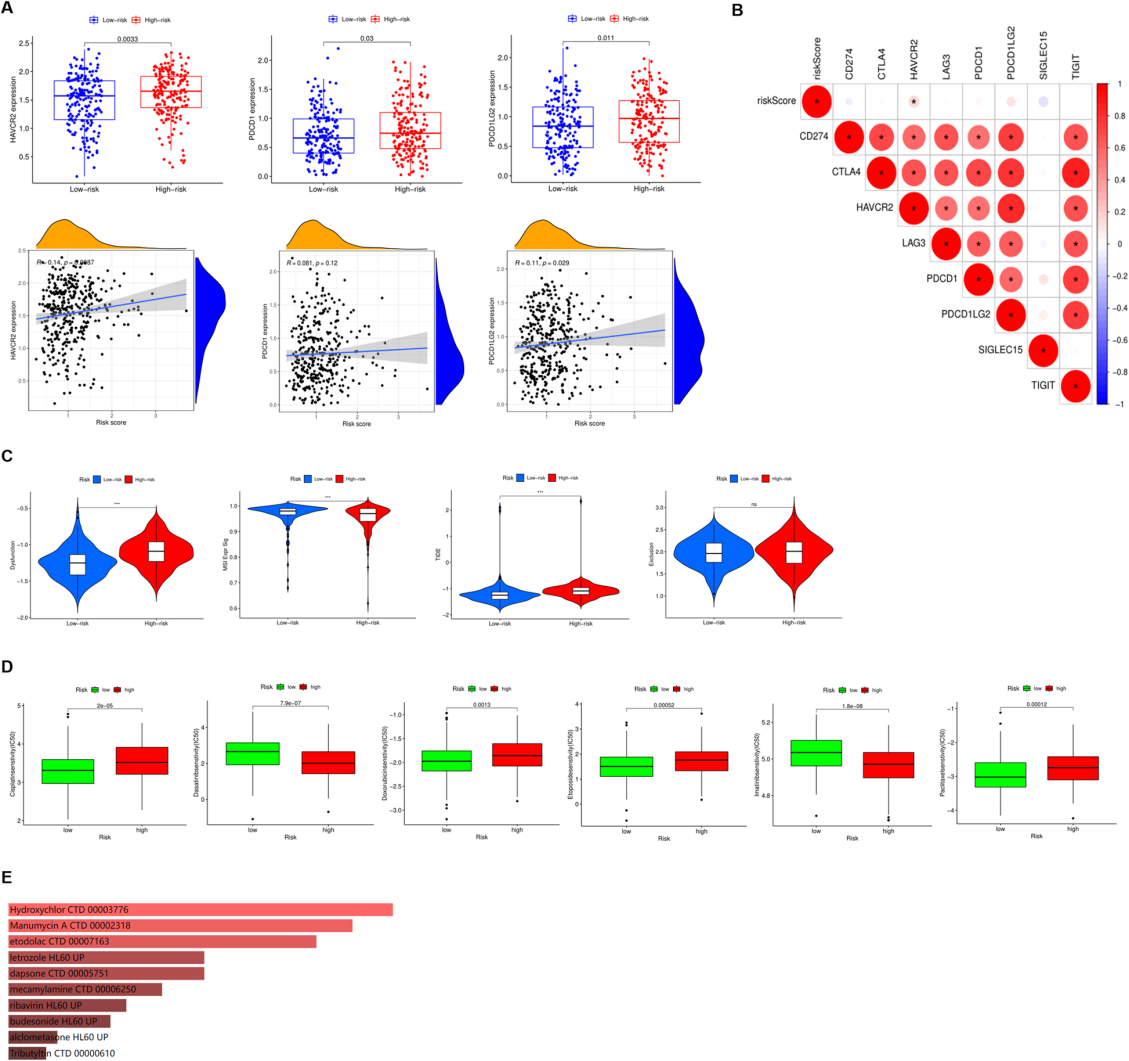
Evaluation of the immunotherapy and chemotherapy between the two groups. **A** Comparison of the expression of immune checkpoint molecules between the two groups. **B** Correlation analysis between the risk score and immune checkpoint molecules. **C** Evaluation of the immunotherapy between the two groups. **D** Evaluation of the chemotherapy between the two groups. **E** Evaluation of the chemotherapy drugs based on the key genes in the model

Immunotherapy is a new promising option for the future treatment of OC. Therefore, we further evaluated the association of immunotherapy with risk score by analyzing the correlation between risk score and expression of immune checkpoint molecules. First, the expression of immune checkpoint molecules in high and low risk groups was compared. The results showed that the expressions of *HAVCR2*, *PDCD1*, and *PDCD1LG2* were significantly higher in the high-risk group than in the low-risk group, and their expression levels were also positively correlated with the risk score (Fig. 7A). Correlation analysis revealed a close association between checkpoint molecules, and a clear positive correlation between *HAVCR2* and risk score (Fig. 7B). Next, for a comprehensive assessment, we further included the TIDE score, T-cell dysfunction score, and T-cell rejection score, which were more accurate biomarkers in our analysis. As expected, low-risk patients were characterized by significantly lower TIDE scores, lower T-cell dysfunction scores, and higher MSI scores, with no significant difference in T-cell exclusion scores, suggesting that low-risk patients tended to be more sensitive to immunotherapy response (Fig. 7C). Chemotherapy drugs are still the main and most effective treatment for OC. We evaluated the responsiveness of high and low risk groups to different chemotherapy drugs, and found that the IC50 of Cisplatin, Dasatinib, Doxorubicin, Etoposide, Imatinib, and Paclitaxel in the low risk group were significantly lower than the high-risk group, indicating that the low-risk group was more responsive to chemotherapy drugs, indicating a better prognosis (Fig. 7D). Eventually, ENRICHR was utilized to explore potential drugs based on the key genes in the model, and the results indicated that Hycroxychlor, Manumycin, etodolac, letrozole, dapsone, mecamylamine, ribavirin, budesonide, alclometasone and Tributyltin were favorable drugs (Fig. 7E).

### Tumor mutation analysis

Tumor gene mutation was closely related to tumor development and poor prognosis, so we calculated the gene mutation frequency of patients. It was found that 420 of the 436 clinical samples had gene mutations. The top three genes with the highest mutation frequency were *TP53*, *TTN* and *MUC16*, and the most common type of mutation was missense mutation (Fig. S11A). In order to analyze the mutation spectrum between high- and low-risk groups, we performed mutation analysis on high-risk and low-risk patients, respectively, and found that 125 of the 134 patients in the low-risk group had gene mutations, and the genes with the top three mutation frequencies were *TP53*, *TTN* and *MUC16* (Fig. S11B), 127 of 138 patients in the high-risk group had gene mutations, and the top three genes with mutation frequencies were *TP53*, *TTN* and *CSMD3* (Fig. S11C). At the same time, we studied the mutation status of risk model genes in cBioportal. First, the most common type of mutation in OC was amplification. The highest mutation frequency in model genes was *AP1M2*, followed by *COL16A1*, and the third was *SPOCK2*. The specific mutation types for each gene were placed in the histogram (Fig. S11D). At the same time, the mutation positions and types of key genes are shown in Fig. S11E. Copy number variation (CNV) is the deletion or duplication of genomic fragments of more than 1 kb caused by genome rearrangement. Genomic alterations and CNVs are hallmarks of cancer, and different kinds of cancers are characterized as unique aberrations, providing clues to the cause and prognosis of the disease. Therefore, the CNV spectrum of the key genes was also explored and the results illustrated that *AP1M2*, *SPOCK2*, *ENOSF1*, *GRB7* were enriched in higher gain of copies of a genomic DNA region, while *COL16A1*, *RIPK4* and *CXCR4* were opposite. Eventually, an overview of the occurrence of CNVs in different chromosomes of these core genes was also described (Fig. S11F). TMB is the total number of gene mutations in tumor tissue, and high TMB may indicate better immunotherapy effect. We therefore compared the TMB values in the high and low risk groups, but there was no significant difference between the two groups (Fig. S11G). Finally, we integrated risk scores with TMB and immune cell correlations in a circle plot, illustrating the close connection between TMB and immune cells (Fig. S11H).

### Validation of model gene expression and survival

**Fig. 8:**
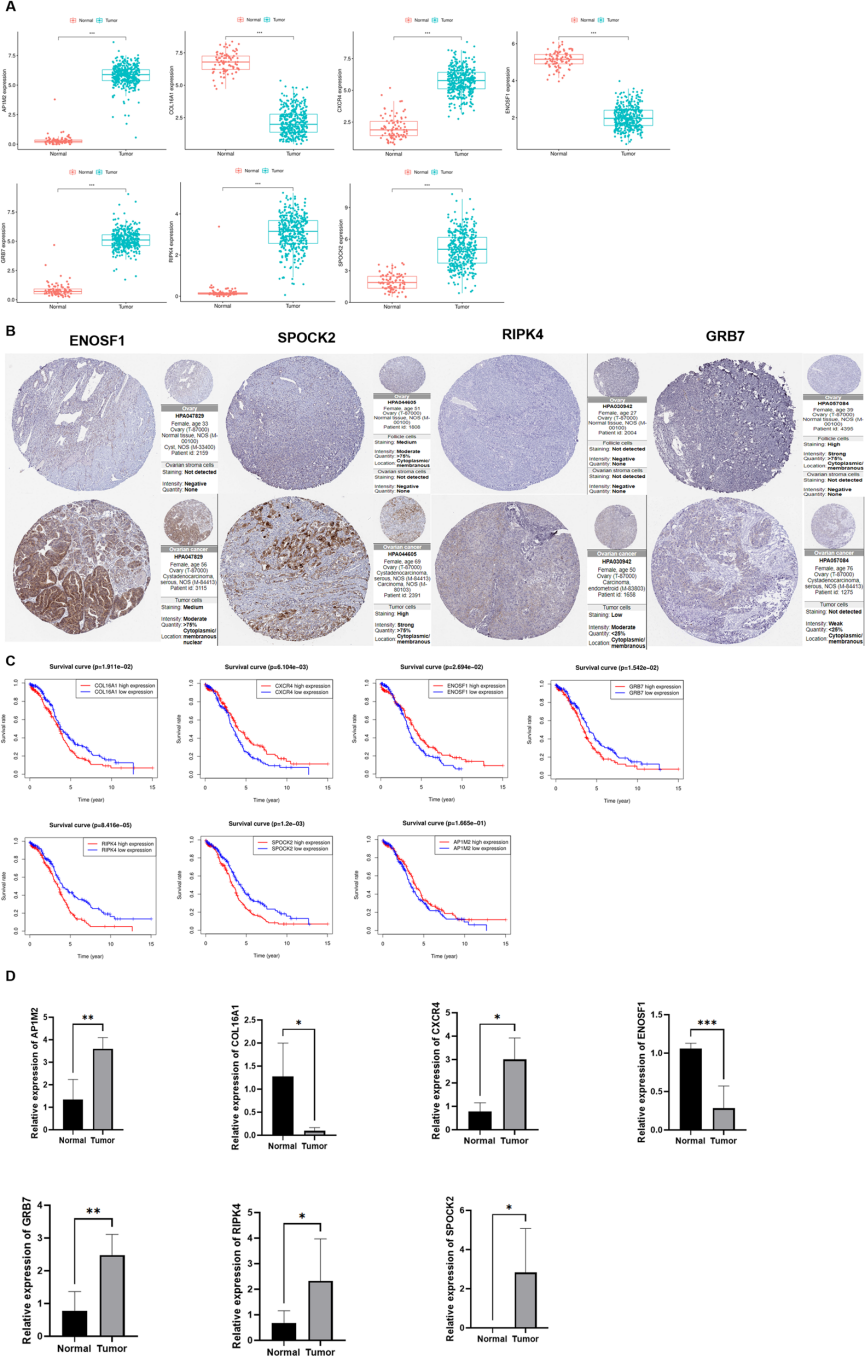
Validation of model gene expression and survival. **A** Expression of the key genes between cancer and normal tissue. **B** The protein expression profiles of the key genes in human protein atlas. **C** Survival analysis for the key genes. **D** The mRNA expression level of the key genes in the clinical samples

To investigate the role of model genes in OC, we performed differential expression analysis to identify their expression, and found that *AP1M2*, *CXCR4*, *GRB7*, *RIPK4*, and *SPOCK2* were significantly increased in OC, while *COL16A1* and *ENOSF1* were significantly decreased (Fig. 8A). Meanwhile, the protein expression profiles of the key genes were also studied, which were similar with precious results (Fig. 8B). Further survival analysis also showed that high expression of *CXCR4* and *ENOSF1* predicted better prognosis, while patients with low expression of *GRB7*, *RIPK4*, *SPOCK2*, and *COL16A1* had better prognosis (Fig. 8C). Besides, we also investigated and verified the mRNA expression of the key genes via qRT-PCR, and the results indicated that the expression of *AP1M2*, *CXCR4*, *GRB7*, *RIPK4*, and *SPOCK2* were upregulated in OC, while *COL16A1* and *ENOSF1* were downregulated, which was similar with the previous results (Fig. 8D).

## Discussion

OC is one of the most common malignant tumors in the female reproductive system, and its mortality rate ranks first among gynecological malignant tumors. In 2020, the U.S. is expected to have 21,750 new cases of ovarian cancer and 13,940 deaths [1]. Although the current diagnosis and treatment methods have gradually improved and the survival and prognosis of patients have been improved to a certain extent, the characteristics of OC are highly heterogeneous and the molecular mechanisms of the occurrence and development of different types of OC are different, thus the responsiveness of different treatment methods is also quite different. Accurate and individualized predictive biomarkers at the molecular level are of great significance for the clinical diagnosis, treatment and prognosis evaluation of OC.

In this study, we adopted a scRNA-seq dataset to characterize OC heterogeneity. OC cells with different differentiation states were predicted into three subpopulations based on cell trajectory analysis, and subpopulation-dependent molecular phenotypes were identified. Based on the DEGs identified in the three subgroups, molecular function analysis showed that State1 and State2 were enriched in negative regulation of cell adhesion, cell adhesion mediator activity, regulation of cell-cell adhesion; State2 was enriched in T cell activation, while State3 was enriched in extracellular matrix organization, collagen-containing extracellular matrix, extracellular matrix structural constituent. These results suggested that different cellular differentiation trajectories could reflect the heterogeneity of OC, which was closely related to cell adhesion and immune pathways.

Subsequently, based on the DEGs identified above, we performed consensus clustering analysis in the TCGA dataset, and the patients were well divided into two clusters, C1 and C2. Survival analysis confirmed that patients in the C1 had better survival prognosis. Further study of the infiltration ratio of immune cells in the two clusters found that T cells CD4 memory resting, Monocytes, Eosinophil and Neutrophils were significantly higher in the C2 than in the C1, which was closely related to poor prognosis. Besides, the expression of immune checkpoints was significantly higher in the C2 than in the C1, and was significantly associated with immunotherapy responsiveness and survival prognosis.

We further investigated the potential roles of these signature genes in OC. Based on TCGA-OC data, we performed WGCNA analysis to identify 574 key genes in the core module, and differential analysis further screened a total of 156 genes closely related to OC. Based on these hub genes, the expression profiles in the TCGA and GEO datasets were normalized to remove batch effects, and TCGA was set as the training set and GEO as the validation set. Therefore, we first identified 11 genes associated with prognosis (*LTBP3*, *COL16A1*, *ENOSF1*, *SPOCK2*, *EPCAM*, *JUP*, *AP1M2*, *GRB7*, *RIPK4*, *CXCR4*, *SRP9*) by univariate COX analysis in the TCGA dataset. LASSO cox regression analysis constructed a prognostic risk model consisting of 7 genes *COL16A1*, *ENOSF1*, *SPOCK2*, *AP1M2*, *GRB7*, *RIPK4*, *CXCR4*. Based on the formula of risk score, patients in the training set were divided into high and low risk groups. Survival analysis indicated that patients in the low risk group had better prognosis, and subsequent independent prognostic analysis further illustrated that the risk score, similar to other clinical characteristics such as age, was independent prognostic risk factors in OC. Not only that, the ROC, DCA curves and the C-index comparison with other established models confirmed that our model had excellent predictive performance, which was also well illustrated in the validation set.

Recently, there has been increasing evidence that the key genes in the model was a potential target of OC. *COL16A1*, secreted and synthesized by uterine stromal cells, affected collagen breakdown and absorption [7]. *COL16A1* was identified as a risk model in a prognostic model of OC, and its expression was significantly increased in ovarian cancer tissue and predicted poor prognosis [8].

*ENOSF1* encoded a mitochondrial enzyme to convert L-fuconate to 2-keto-3-deoxy-L-fuconate. The expression level of *ENOSF1* in the serum of gastric cancer patients was significantly higher than that of healthy controls, which could be used as a potential gastric cancer serum biomarker [9]. However, there were few studies of this gene in cancer at present. In this study, the expression of *ENOSF1* was significantly reduced in OC tissue, and low expression predicted a good prognosis, indicating that *ENOSF1* acted as a tumor suppressor gene in OC. Therefore, the mechanism of this gene in the occurrence and development of OC could be further explored in the future, providing a new direction for future targeted therapy.

*SPOCK2*, a secreted protein acidic and cysteine-rich gene with osteonectin, cwcv and kazal-like domains Proteoglycan 2 (*SPOCK2*), encoded a protein that binded to glycosaminoglycans to form the extracellular matrix and playd an important role in cell invasion and metastasis. A study on endometrial cancer (EC) showed that the expression level of *SPOCK2* in EC was significantly lower than that in normal endometrial tissue, and the lack of *SPOCK2* protein expression was associated with distant metastasis and myometrial invasion, which regulated the biological behavior of cancer cells, thus promoting the advance of EC [10]. In addition, the role of *SPOCK2* in OC had also been studied. The expression of *SPOCK2* in advanced OC was significantly higher than that in early OC and indicated poor prognosis of patients. In-depth study of its specific mechanism found that miR-363-3p-*SPOCK2* Axis ws involved in the regulation of the actin cytoskeleton and inhibits OC progression, which was consistent with our findings [11].

*AP1M2*, belongs to adnectin-associated adaptor protein complex 1 and functions in the anti-Golgi network and protein sorting in the endothelium. A pan-cancer analysis of *AP1M2* showed that *AP1M2* was abundantly expressed in various cancers, and its expression level was positively correlated with the prognosis of tumor patients. By studying the effect of *AP1M2* on the clinical prognosis and immune infiltration of tumor patients, it was found that the expression of *AP1M2* in breast invasive cancer was associated with the overall survival of patients and the infiltration levels of macrophages, dendritic cells, and T cells (CD4^+^ and CD8^+^) and B cells. In addition, *AP1M2* expression was positively correlated with tumor immune neoantigens and microsatellite instability in invasive breast carcinoma [12].

Growth factor receptor binding protein 7 (*GRB7*) was a member of the *GRB7* signaling protein family, which was involved in the erythrocytic leukemia virus oncogene homolog receptor family, platelet-derived growth factor receptor, insulin receptor, and RAS-GTP enzymes and other important regulators of cell growth, suggesting that *GRB7* was involved in cell survival and growth [13]. *GRB7* was involved in the occurrence and development of various malignant tumors. A study on cervical cancer found that *GRB7* protein expression was significantly higher in cancer tissues than in non-cancer tissues, and was associated with age, tumor size, serosal invasion, degree of differentiation, tumor stage, early or advanced stage, and lymph node metastasis. Step survival analysis showed that the overall survival rate of patients with positive expression of *GRB7* protein was lower than that of patients with no expression of *GRB7* [14]. In addition, *GRB7* mRNA was upregulated in bladder cancer samples and Overexpression of *GRB7* significantly promoted bladder cancer proliferation and tumorigenesis [15]. Next, *GRB7* has also been studied in OC. Knockout of *GRB7* also significantly inhibited about 40% of cell proliferation (*P* = 0.0024), 95% of cell migration ability (*P* < 0.0001), and 45% of cell invasion ability, suggesting that oncogenesis of *GRB7* [16]. This was also consistent with the results of this study, the expression of *GRB7* was significantly increased in ovarian cancer tissues and high expression predicted poor prognosis.

*RIPK4*, receptor-interacting protein serine/threonine kinase 4, is aberrantly expressed in multiple cancer types and is a key member of the receptor-interacting protein group, which has been studied in detail in OC [17]. The expression level of *RIPK4* in OC tissues and cells is higher than that in normal ovarian tissues and cells. Down-regulation of *RIPK4* expression could inhibit EMT in OC by inhibiting IL-6, which was characterized was a prognostic marker for OC [18]. Meanwhile, Liu et al. demonstrated that *RIPK4* could serve as a potential prognostic molecular marker for poor survival in OC patients. Furthermore, *RIPK4* was also associated with tumor metastasis and is involved in the regulation of the Wnt signaling pathway [19].

*CXCR4*, a member of the C-X-C chemokine receptor family, is associated with a variety of cancers, and high *CXCR4* expression in esophageal, gastric, and colorectal cancers predicts poor prognosis [20]. Higher *CXCR4* expression in OC was associated with worse progression-free survival (HR, 8.48; 95% CI: 2.13-33.70; *P* = 0.002) and lower OS [21]. The *CXCL12*/*CXCR4* axis, together with a variety of other factors, regulated tumor growth and metastasis in OC [22] and the concomitant expression of *CD40* and *CXCR4* in OC was strongly associated with pelvic metastasis [23]. Knockdown of *CXCR4* in vitro reduced cell proliferation while increasing apoptosis and chemosensitivity in EOC cell lines [24].

In terms of the role of key genes in the differentiation of OC, *COL16A1* increased in cluster 4 and played an important role in state2; *ENOSF1*, *GRB7*, *RIPK4* increased in cluster 11 and played an important role in state1, *SPOCK2* increased in cluster 6 and played an important role in state1, *AP1M2* was increased in cluster 8 and played an important role in state1, and *CXCR4* was significantly increased in cluster 0 and played an important role in state2, mediating different differentiation progression outcomes.

Previous studies have shown that the heterogeneity of OC also affects the tumor microenvironment and the level of immune cell infiltration, thus influencing the responsiveness of immunotherapy. Therefore, we next analyzed the composition of the tumor microenvironment between the high and low risk groups, and found that the levels of Macrophages, Neutrophils, T helper cells, and TIL cells in the high- risk group were significantly higher than those in the low risk group, while NK cells were opposite. The results showed that the anti-tumor immunity of patients in the high-risk group was low, while the infiltration level of anti-tumor immune cells in the low-risk group was high, which to a certain extent also revealed that the patients in the low-risk group had a better prognosis. Based on the above observations, we further explored differences in sensitivity to chemotherapeutic drugs and immunotherapy in high- and low-risk groups. Amazing discovery,

The IC50s of Cisplatin, Dasatinib, Doxorubicin, Etoposide, Imatinib, and Paclitaxel in the low-risk group were significantly lower than those in the high-risk group, indicating that the low-risk group was more sensitive to chemotherapeutic drugs, which also suggested a better prognosis. As for immunotherapy, the expression of immune checkpoint molecules in the high-risk group was significantly higher than that in the low-risk group. Immune checkpoint molecules were regulatory molecules that inhibited the function of immune cells and the anti-tumor immune response in the immune system, and ultimately led to immune escape. Previous studies have also described that the expression of immune checkpoint molecules was abnormally expressed in OC. Current immunotherapy for OC was also mainly based on immune checkpoint blockade strategies. The TIDE score was applied to assess potential responsiveness to immunotherapy in high- and low-risk groups. The results were consistent with the previous results, and the TIDE score of patients in the low-risk group was significantly lower than that in the high-risk group, and a low TIDE score also predicted a more sensitive immunotherapy effect and a better survival prognosis.

In conclusion, in this study, we characterized OC heterogeneity using single-cell sequencing data, identified DEGs in different differentiation clusters, and constructed a 7-gene risk model based on the DEGs, eventually validating the high-efficiency and sensitive performance of the model. In addition, we also found that there were significant differences in the tumor microenvironment and immunotherapy response of patients in the high- and low-risk groups, which also illustrated that the risk model constructed was closely associated with the tumor immune status. Therefore, our study provided a new risk model for predicting OC prognosis and elucidated OC heterogeneity and its relationship to the immune microenvironment to some extent.

## Supplementary Information

**Additional file 1: Fig. S1.** Analysis of single-cell RNA sequencing in GSE154600. A. Post quality control filtering of each sequenced cell, which was plotted in violin plots to display their number of nFeature_RNA, nCount_RNA, percent_HB, percent_MT and percent_Ribosome. B. Correlation analysis between sequencing depth and mitochondrial gene sequences, ribosome and total intracellular sequences. C. 19,862 non-variable genes and 2000 variable genes were analyzed. D. PCA based on scRNA-seq data. E. 16 PCs were identified as the criteria of P < 0.05. F. Heatmap illustrated the expression patterns of the top ten markers in individual cells of each cluster by Seurat analysis. G. Cells were clustered into 16 types via tSNE analysis and annotation of different cell clusters via Monocle2 package. **Fig. S2.** Functional analysis of three subsets based on DEGs. A. GO analyses for three subsets. B. KEGG enrichment analyses for three subsets. **Fig. S3.** Validation of the risk signature in GSE23554. A. PCA and tSNE analyses of patients in the testing set. B. Distribution of the risk score and survival status of patients in the testing set. C. Kaplan-Meier analysis between the low-risk group and the high-risk group in the testing set. D. ROC curves for predicting 1-year, 3-year and 5-year OS in the testing set. **Fig. S4.** Validation of the risk signature in GSE26712. A. PCA and tSNE analyses of patients in the testing set. B. Distribution of the risk score and survival status of patients in the testing set. C. Kaplan-Meier analysis between the low-risk group and the high-risk group in the testing set. D. ROC curves for predicting 1-year, 3-year and 5-year OS in the testing set. **Fig. S5.** Validation of the risk signature in GSE51088. A. PCA and tSNE analyses of patients in the testing set. B. Distribution of the risk score and survival status of patients in the testing set. C. Kaplan-Meier analysis between the low-risk group and the high-risk group in the testing set. D. ROC curves for predicting 1-year, 3-year and 5-year OS in the testing set. **Fig. S6.** Independent prognosis analysis and construction of the nomogram. A. Univariate analysis of risk score and clinicopathological characteristics. B. Multivariate analysis of risk score and clinicopathological characteristics. C. Construction of the nomogram and calibration curves of the risk score and clinicopathological characteristics. **Fig. S7.** Comparison between the risk model and other established models. A. Comparison of the C-index between the risk model and other established signature. B. DCA curves illustrated the advantages of the nomogram and other clinical parameters. C. AUC curves illustrated the advantages of the nomogram and other clinical parameters. **Fig. S8.** Independent prognostic analysis between high and low groups. A. Heatmap illustrated the risk score and clinical parameters. B. Correlation analysis between the risk score and clinical parameters. C. Kaplan-Meier analysis for different clinical groups. **Fig. S9.** Kaplan-Meier analysis for different enrichment of immune cells. **Fig. S10.** Kaplan-Meier analysis for different enrichment of immune functions. **Fig. S11.** Tumor mutation spectrum. A. Overview of the tumor mutation spectrum in TCGA dataset. B. Tumor mutation spectrum of patients in low- risk group. C. Tumor mutation spectrum of patients in high- risk group. D. Overview of the tumor mutation spectrum of the key genes. E. The overview of mutations types of the key genes. F. The frequency and distribution of the CNVs of the key genes visualized by circos plot. G. TMB between the low- and high- risk groups. H. Association between the TMB and risk score and immune cells.

## Data Availability

All the data used in this study can be found in TCGA and GEO database. All presented data in this study are available from the corresponding author upon reasonable request.

## Abbreviations

OC: Ovarian cancer
scRNA-seq: Single-cell RNA sequencing
TCGA: The Cancer Genome Atlas
GEO: Gene Expression Omnibus
PCA: Principal component analysis
DEGs: Differentially expressed genes
ssGSEA: Single-sample gene set enrichment analysis
IC50: Inhibitory concentration
TMB: Tumor mutational burden
GO: Gene Ontology
KEGG: Kyoto Encyclopedia of Genes and Genomes
ROC: Receiver operating characteristic
EC: Endometrial cancer
