# Supplementary material for: Integrated analysis of single-cell RNA-seq and bulk RNA-seq unveils heterogeneity and establishes a novel signature for prognosis and tumor immune microenvironment in ovarian cancer
Source: J Ovarian Res. 2023 Jan 16;16:12. doi: 10.1186/s13048-022-01074-1 (PMC9841625; doi:10.1186/s13048-022-01074-1)

**Figure S1. Analysis of single-cell RNA sequencing in GSE154600**

A. Post quality control filtering of each sequenced cell, which was plotted in violin plots to display their number of nFeature_RNA, nCount_RNA, percent_HB, percent_MT and percent_Ribosome.

B. Correlation analysis between sequencing depth and mitochondrial gene sequences, ribosome and total intracellular sequences

C. 19862 non-variable genes and 2000 variable genes were analyzed

D. PCA based on scRNA-seq data

E. 16 PCs were identified as the criteria of *P* < 0.05

F. Heatmap illustrated the expression patterns of the top ten markers in individual cells of each cluster by Seurat analysis

G. Cells were clustered into 16 types via tSNE analysis and annotation of different cell clusters via Monocle2 package.


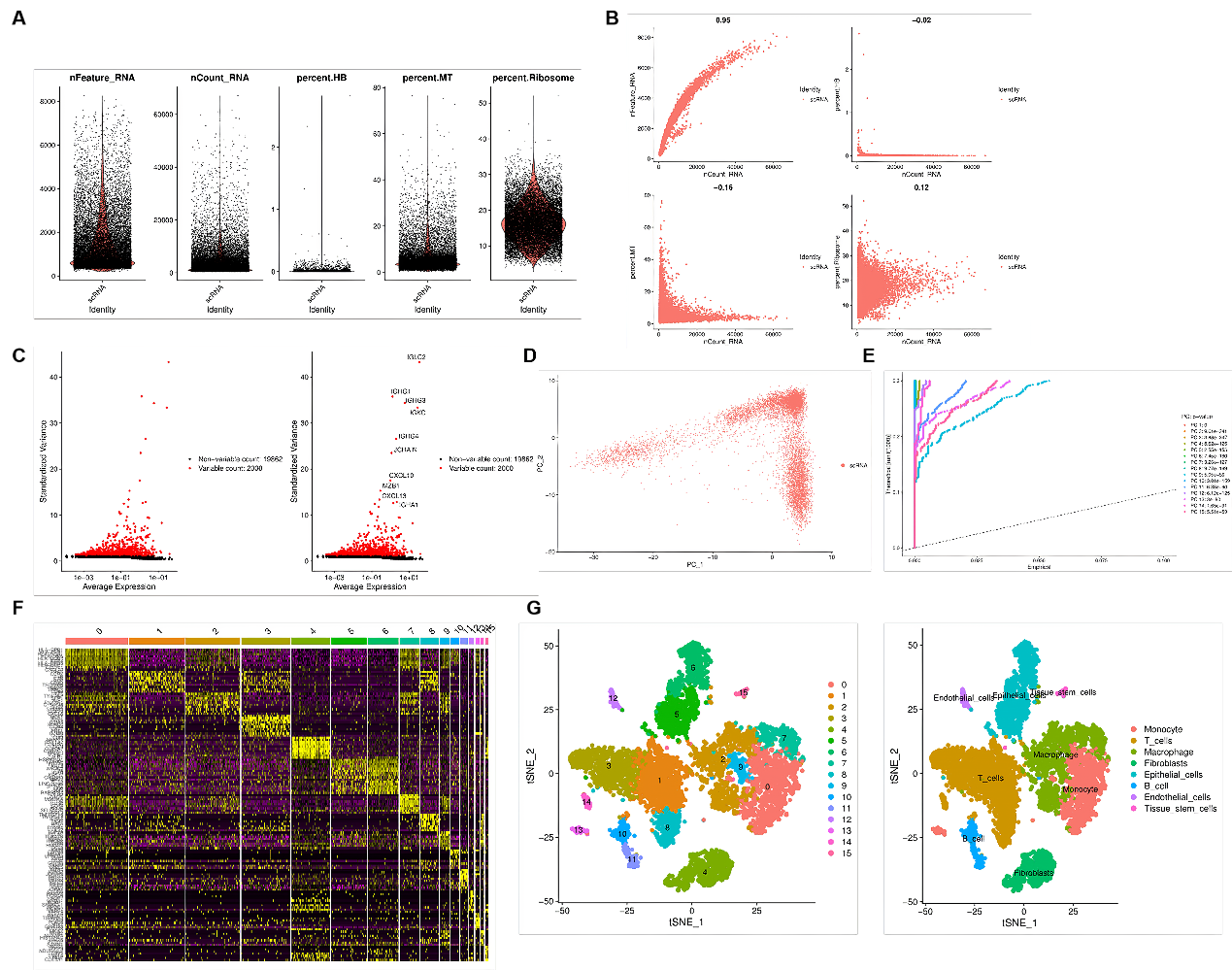


**Figure S2. Functional analysis of three subsets based on DEGs**

A. GO analyses for three subsets

B. KEGG enrichment analyses for three subsets


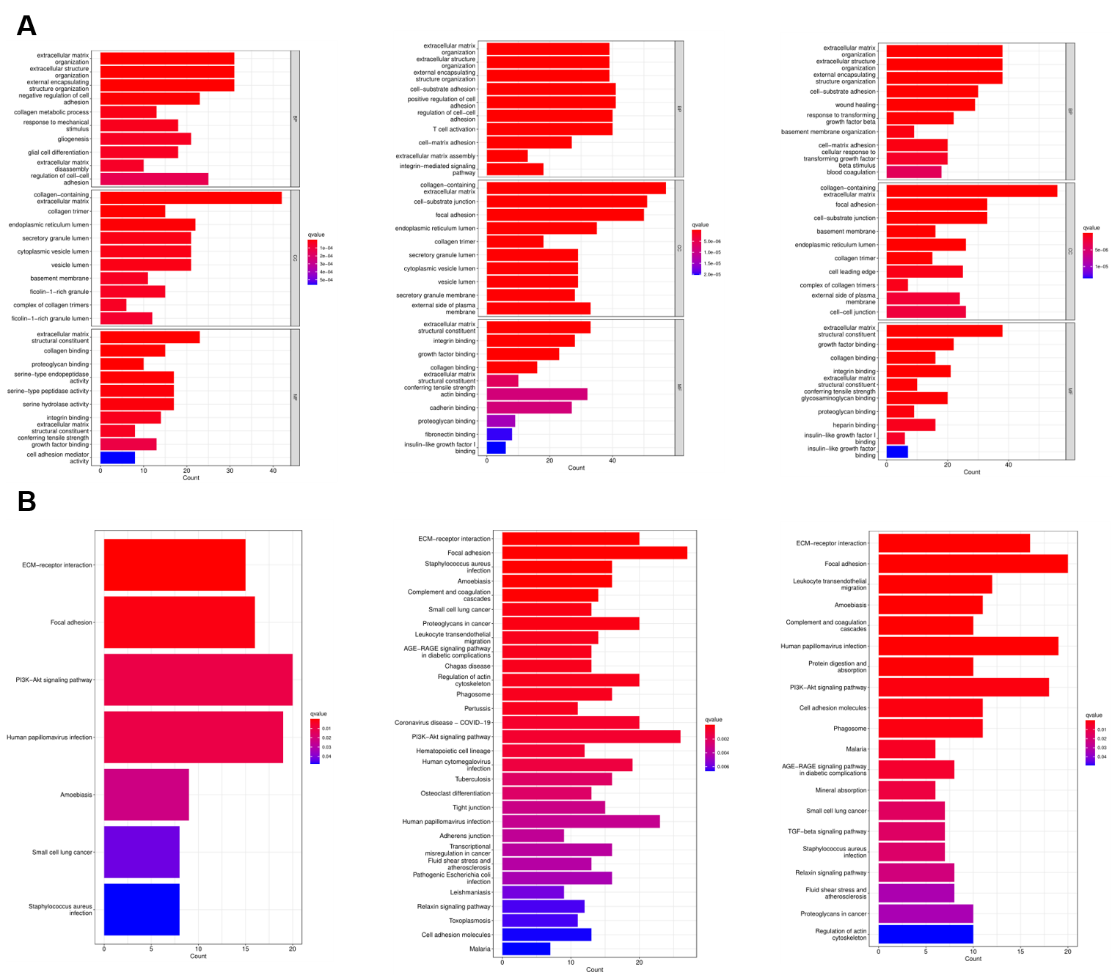


**Figure S3. Validation of the risk signature in GSE23554**

A. PCA and tSNE analyses of patients in the testing set

B. Distribution of the risk score and survival status of patients in the testing set

C. Kaplan-Meier analysis between the low-risk group and the high-risk group in the testing set

D. ROC curves for predicting 1-year, 3-year and 5-year OS in the testing set


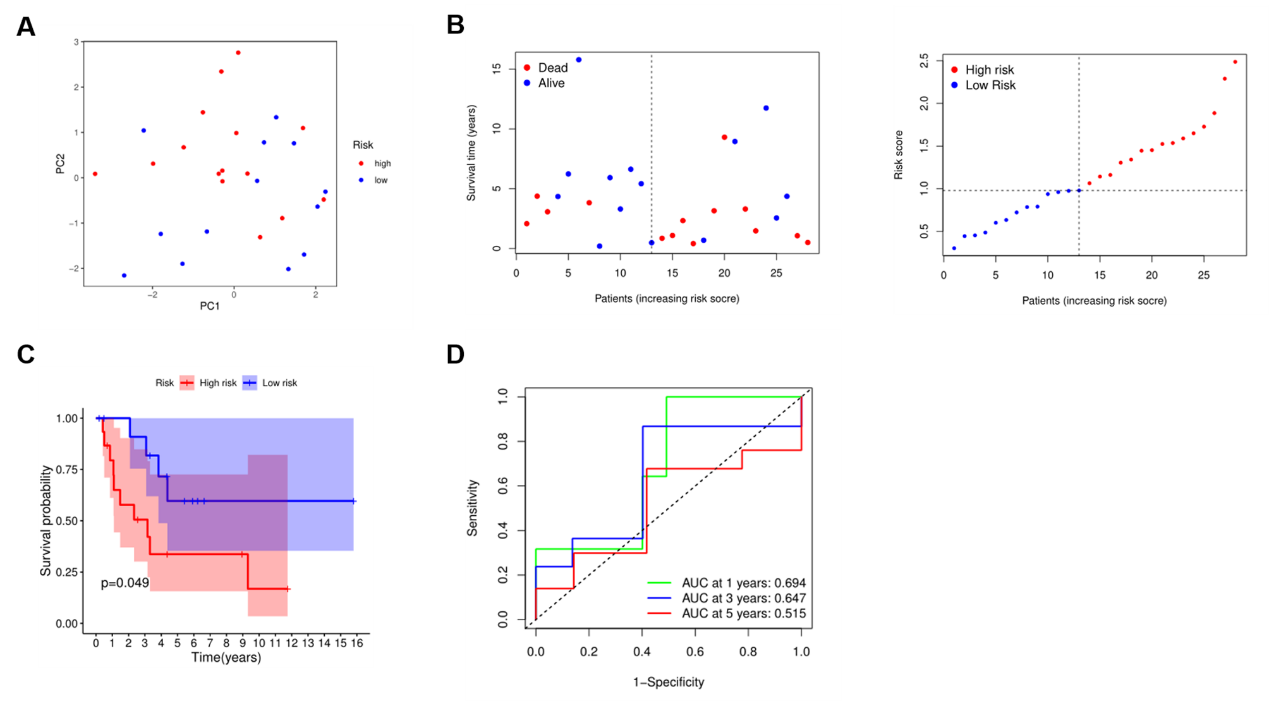


**Figure S4. Validation of the risk signature in GSE26712**

A. PCA and tSNE analyses of patients in the testing set

B. Distribution of the risk score and survival status of patients in the testing set

C. Kaplan-Meier analysis between the low-risk group and the high-risk group in the testing set

D. ROC curves for predicting 1-year, 3-year and 5-year OS in the testing set


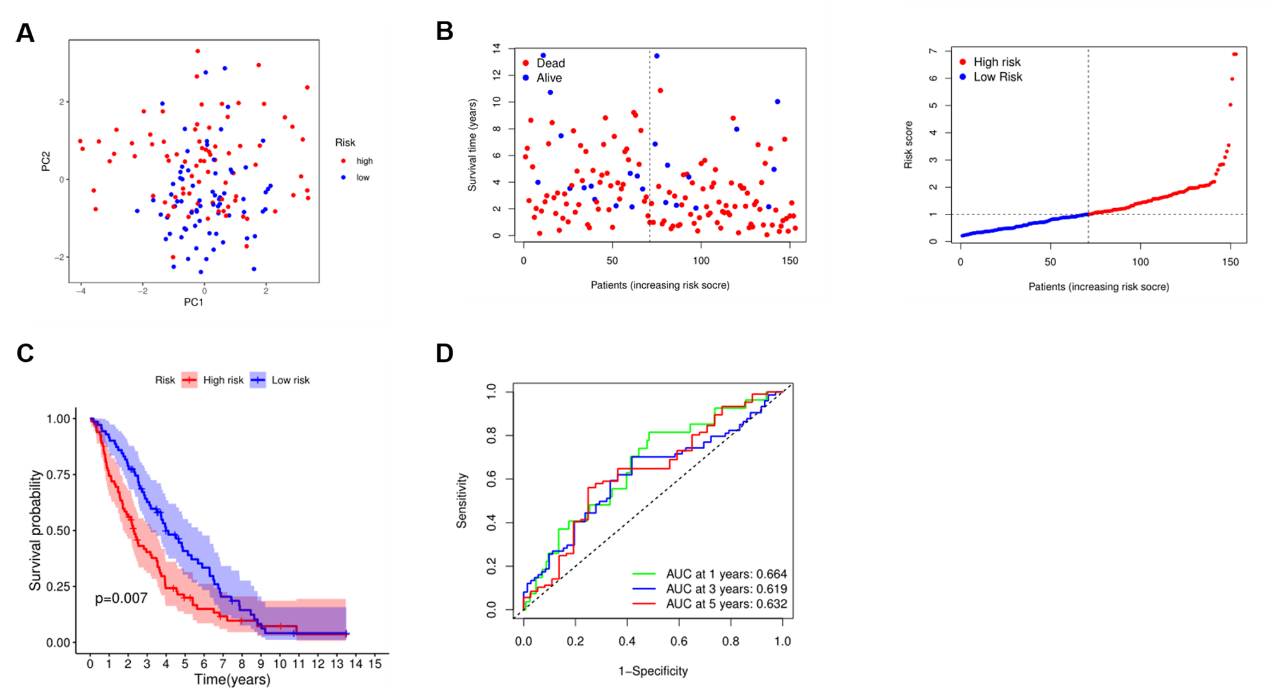


**Figure S5. Validation of the risk signature in GSE51088**

A. PCA and tSNE analyses of patients in the testing set

B. Distribution of the risk score and survival status of patients in the testing set

C. Kaplan-Meier analysis between the low-risk group and the high-risk group in the testing set

D. ROC curves for predicting 1-year, 3-year and 5-year OS in the testing set


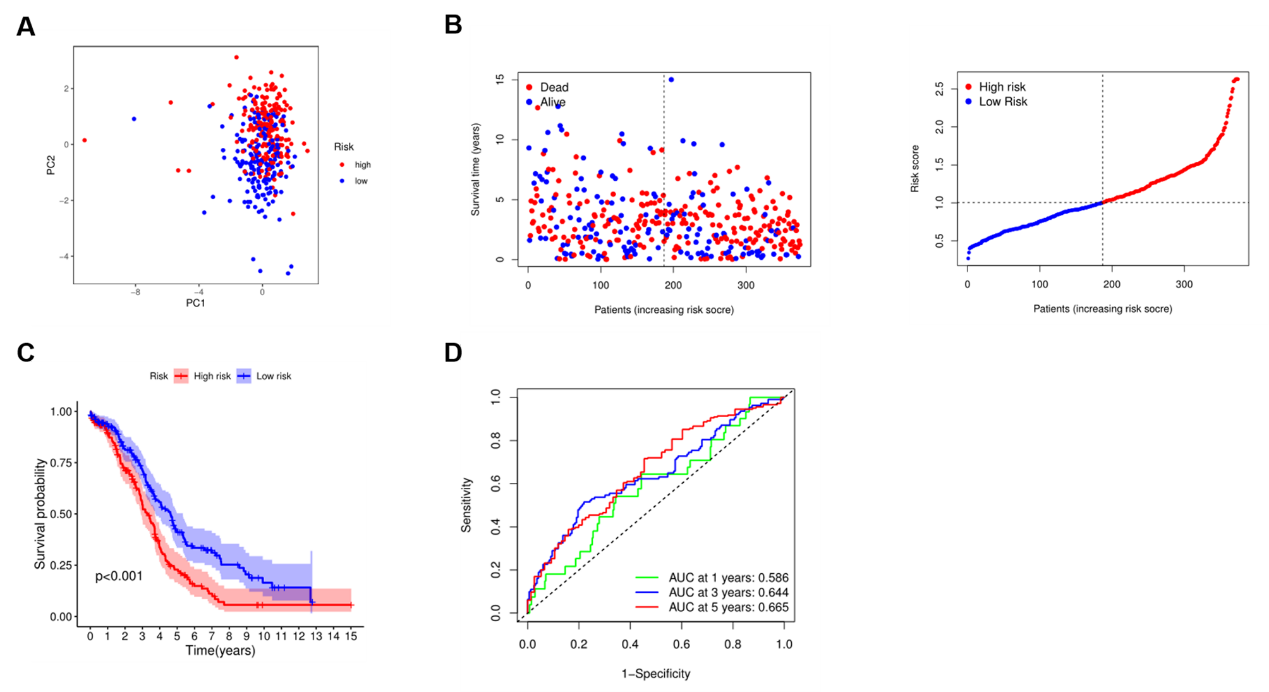


**Figure S6. Independent prognosis analysis and construction of the nomogram**

A. Univariate analysis of risk score and clinicopathological characteristics

B. Multivariate analysis of risk score and clinicopathological characteristics

C. Construction of the nomogram and calibration curves of the risk score and clinicopathological characteristics


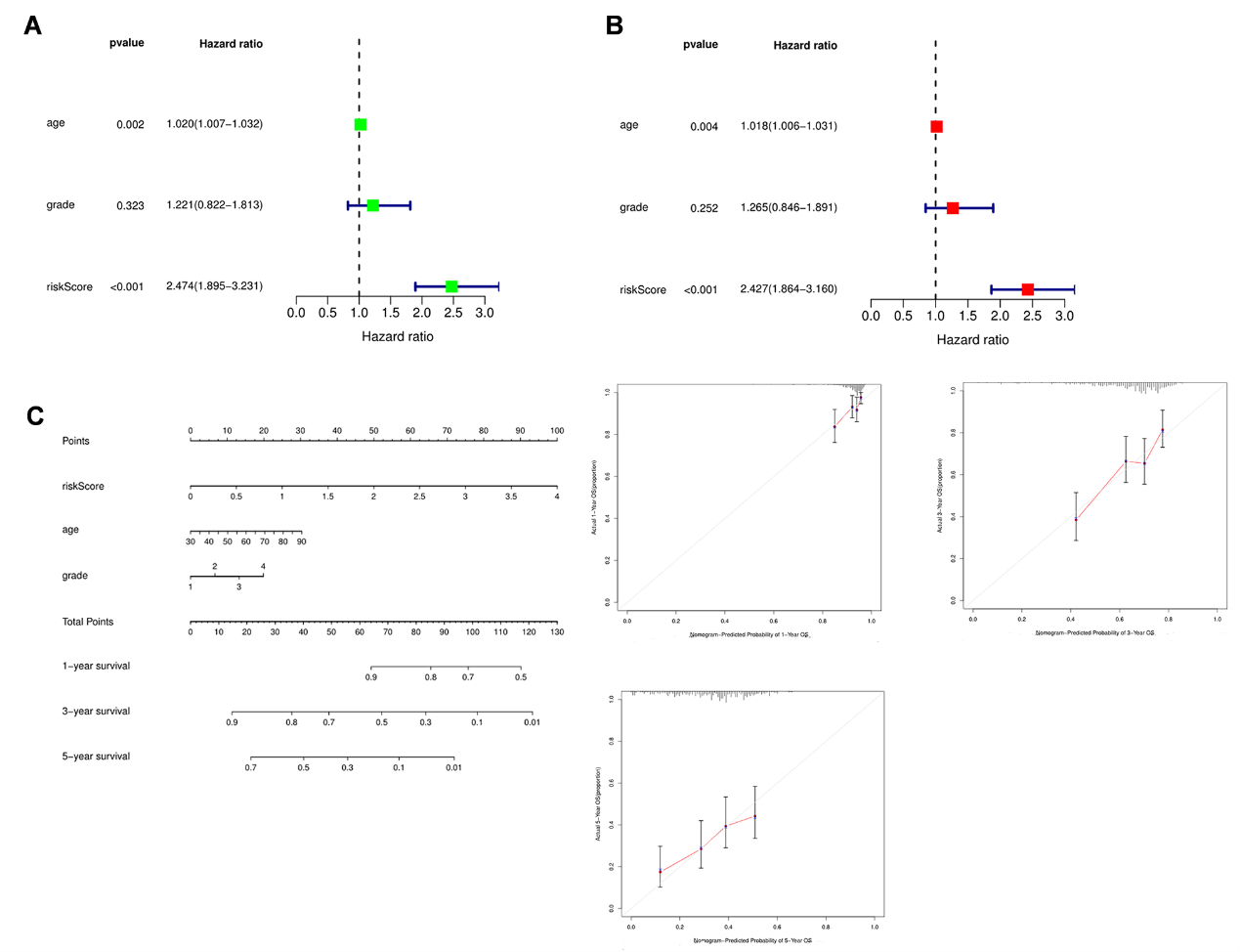


**Figure S7. Comparison between the risk model and other established models**

A. Comparison of the C-index between the risk model and other established signature

B. DCA curves illustrated the advantages of the nomogram and other clinical parameters

C. AUC curves illustrated the advantages of the nomogram and other clinical parameters


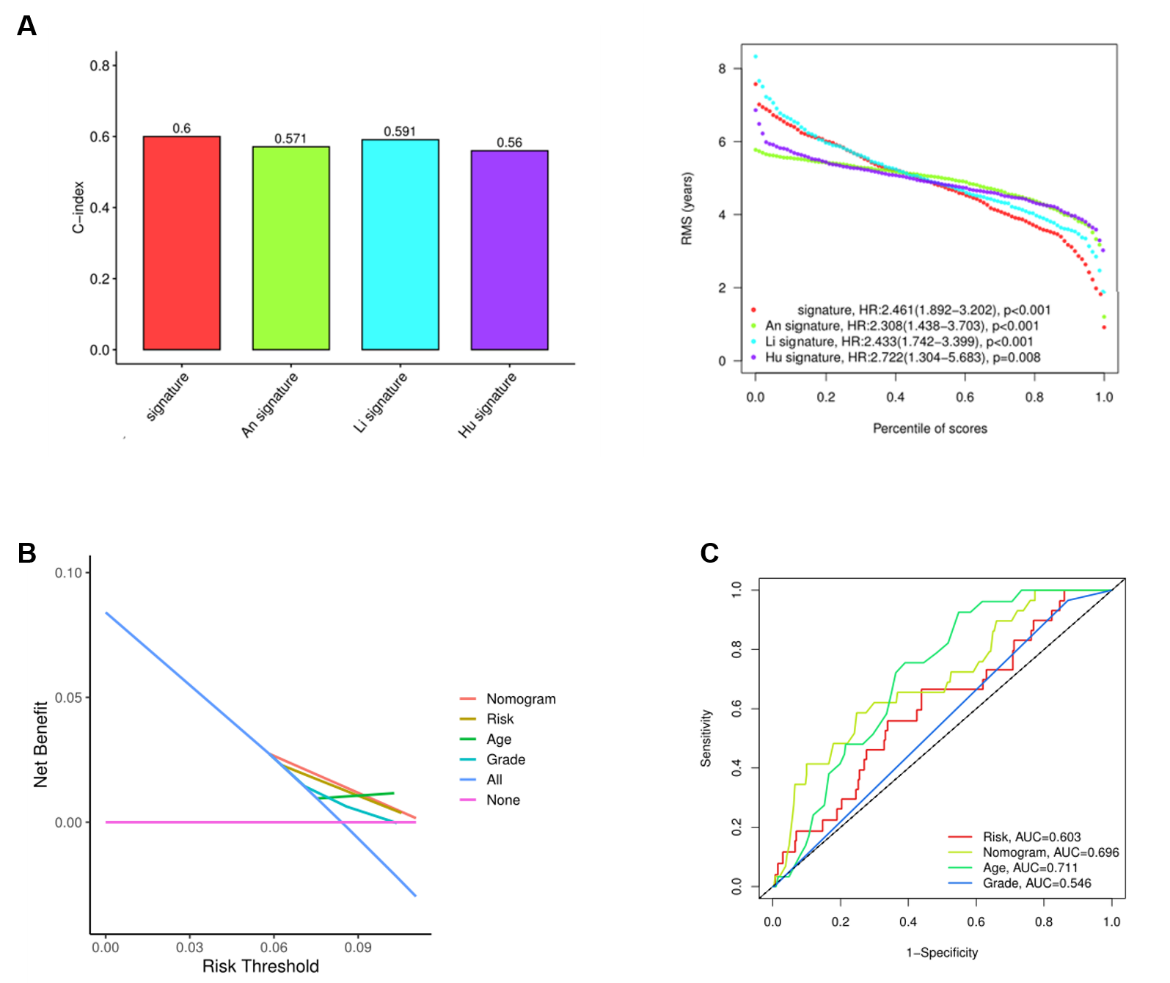


**Figure S8. Independent prognostic analysis between high and low groups**

A. Heatmap illustrated the risk score and clinical parameters

B. Correlation analysis between the risk score and clinical parameters

C. Kaplan-Meier analysis for different clinical groups


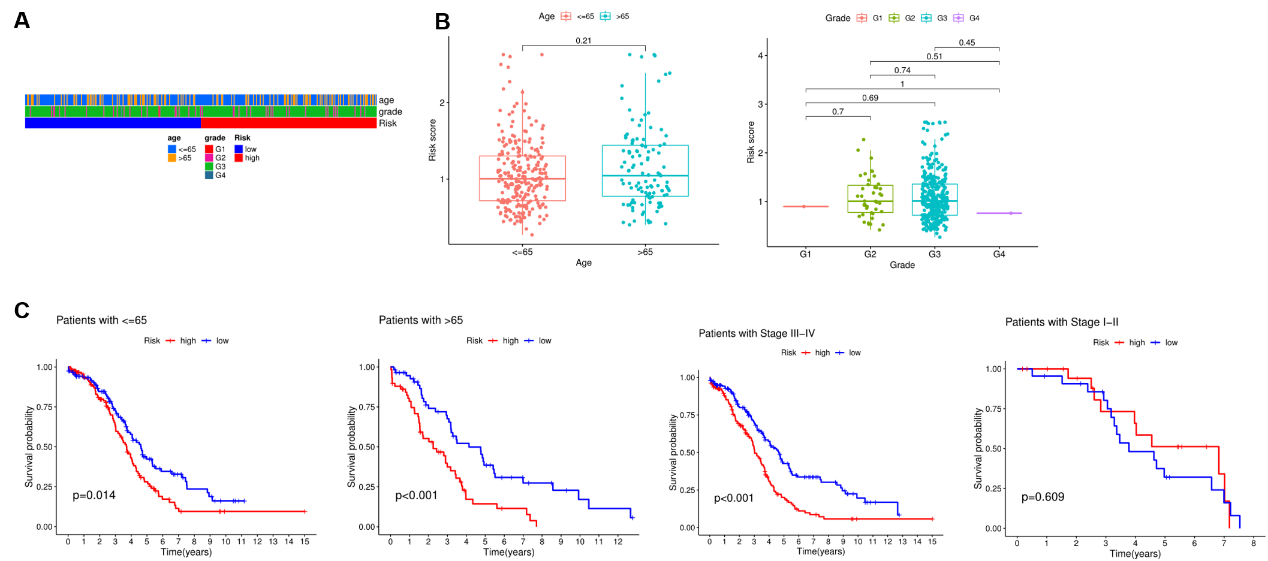


**Figure S9. Kaplan-Meier analysis for different enrichment of immune cells**


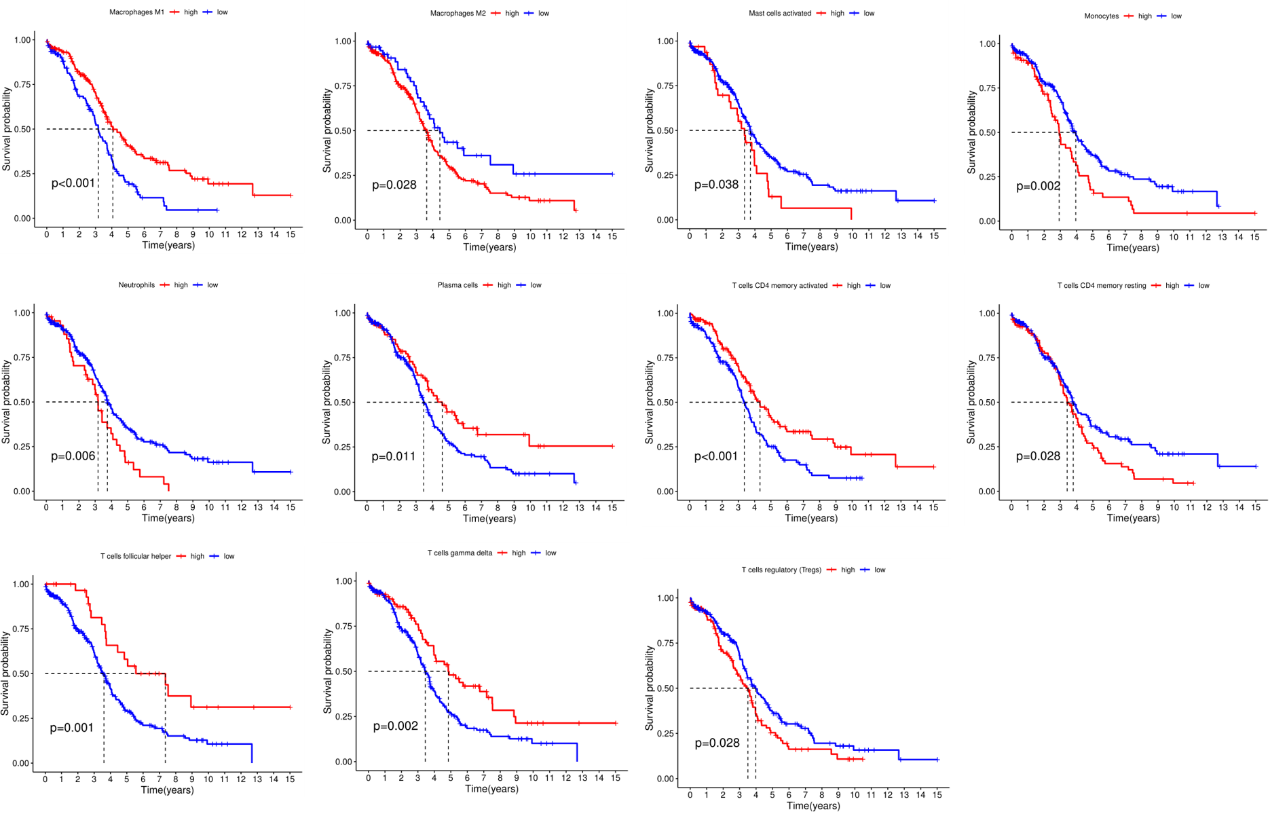


**Figure S10. Kaplan-Meier analysis for different enrichment of immune functions**


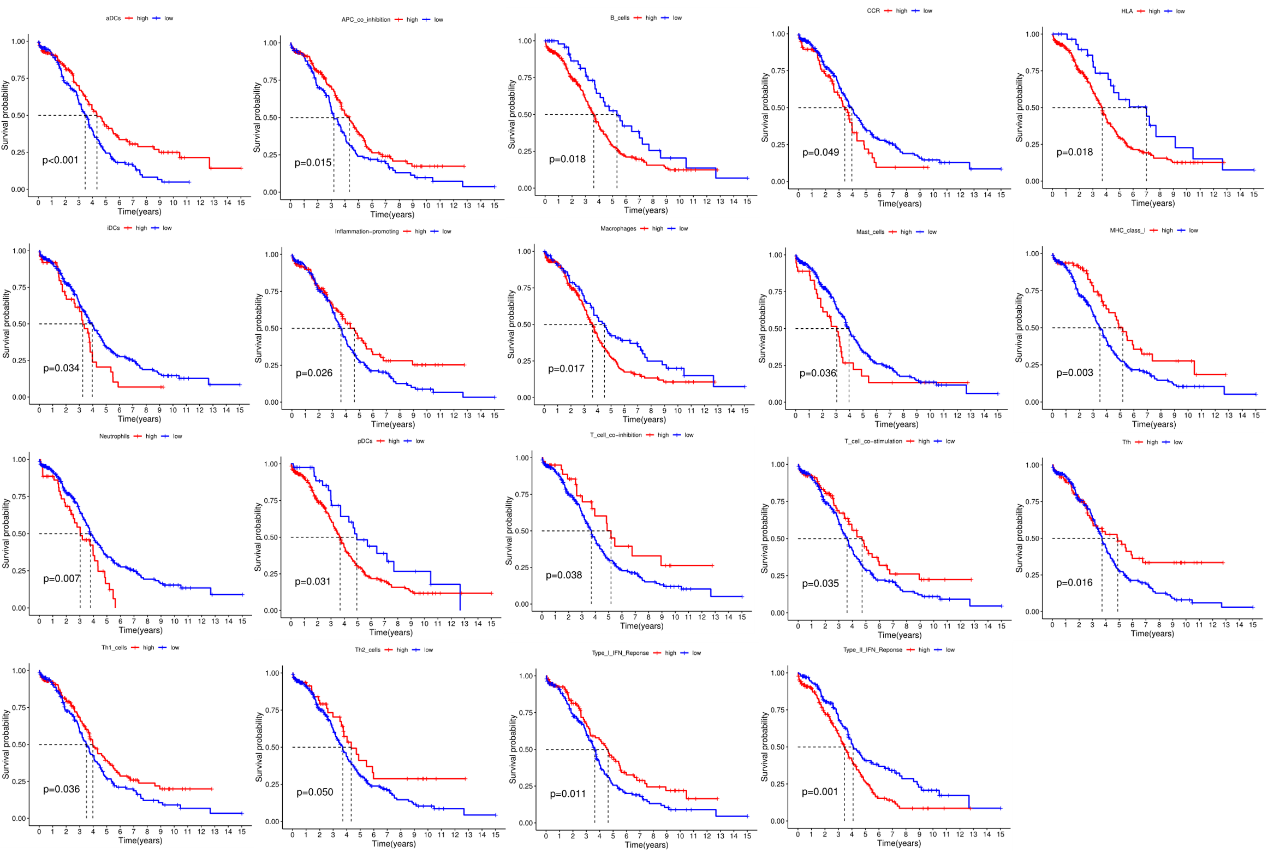


**Figure S11. Tumor mutation spectrum**

A. Overview of the tumor mutation spectrum in TCGA dataset

B. Tumor mutation spectrum of patients in low- risk group

C. Tumor mutation spectrum of patients in high- risk group

D. Overview of the tumor mutation spectrum of the key genes

E. The overview of mutations types of the key genes

F. The frequency and distribution of the CNVs of the key genes visualized by circos plot

G. TMB between the low- and high- risk groups

H. Association between the TMB and risk score and immune cells


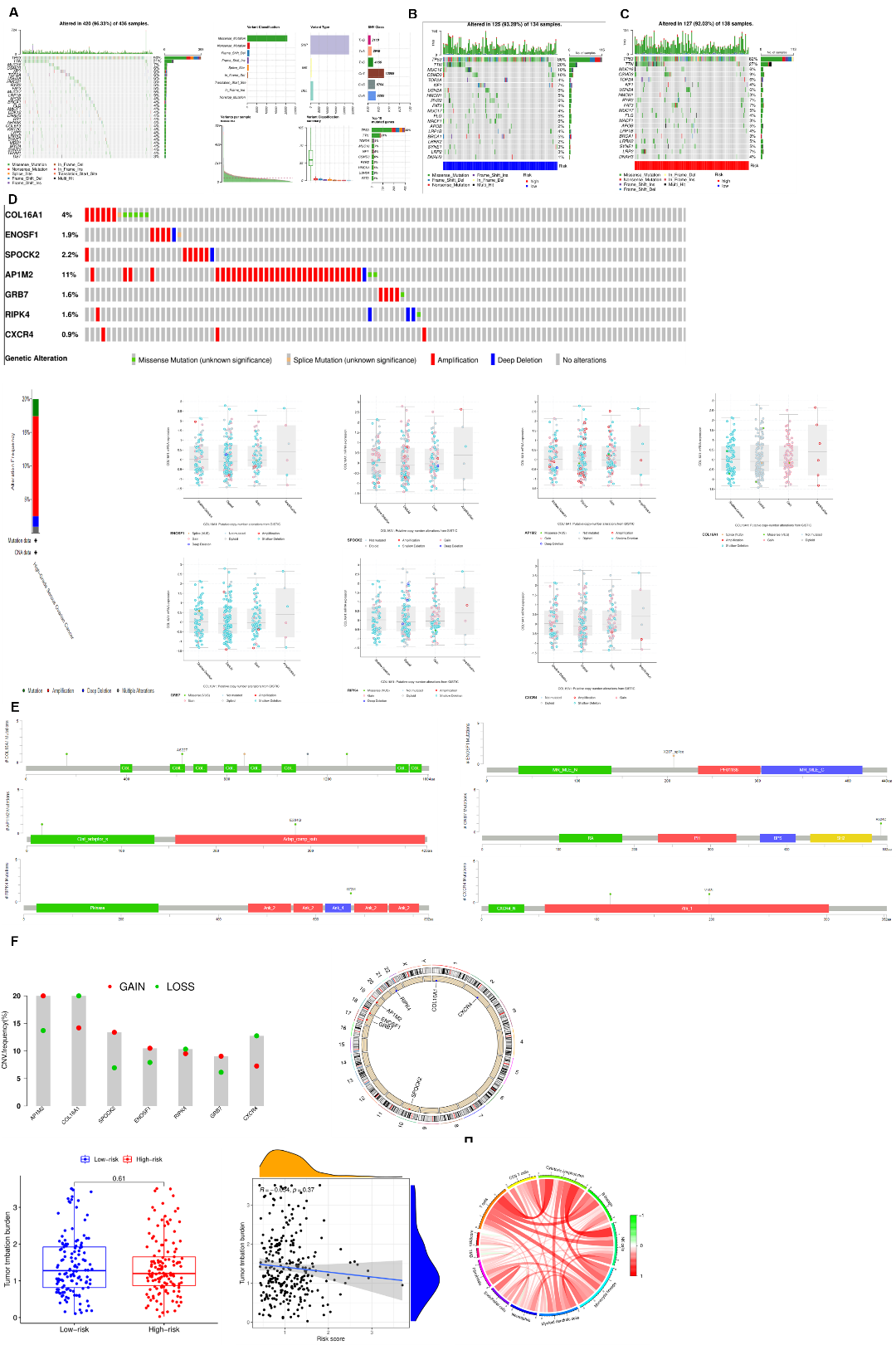

Supplement: Supplementary file 1 — Additional file 1: Fig. S1. Analysis of single-cell RNA sequencing in GSE154600. A. Post quality control filtering of each sequenced cell, which was plotted in violin plots to display their number of nFeature_RNA, nCount_RNA, percent_HB, percent_MT and percent_Ribosome. B. Correlation analysis between sequencing depth and mitochondrial gene sequences, ribosome and total intracellular sequences. C. 19,862 non-variable genes and 2000 variable genes were analyzed. D. PCA based on scRNA-seq data. E. 16 PCs were identified as the criteria of P < 0.05. F. Heatmap illustrated the expression patterns of the top ten markers in individual cells of each cluster by Seurat analysis. G. Cells were clustered into 16 types via tSNE analysis and annotation of different cell clusters via Monocle2 package. Fig. S2. Functional analysis of three subsets based on DEGs. A. GO analyses for three subsets. B. KEGG enrichment analyses for three subsets. Fig. S3. Validation of the risk signature in GSE23554. A. PCA and tSNE analyses of patients in the testing set. B. Distribution of the risk score and survival status of patients in the testing set. C. Kaplan-Meier analysis between the low-risk group and the high-risk group in the testing set. D. ROC curves for predicting 1-year, 3-year and 5-year OS in the testing set. Fig. S4. Validation of the risk signature in GSE26712. A. PCA and tSNE analyses of patients in the testing set. B. Distribution of the risk score and survival status of patients in the testing set. C. Kaplan-Meier analysis between the low-risk group and the high-risk group in the testing set. D. ROC curves for predicting 1-year, 3-year and 5-year OS in the testing set. Fig. S5. Validation of the risk signature in GSE51088. A. PCA and tSNE analyses of patients in the testing set. B. Distribution of the risk score and survival status of patients in the testing set. C. Kaplan-Meier analysis between the low-risk group and the high-risk group in the testing set. D. ROC curves for pre [file 13048_2022_1074_MOESM1_ESM.docx]
